# Allelic variations of *Vrn-1* and *Ppd-1* genes in Japanese wheat varieties reveal the genotype-environment interaction for heading time

**DOI:** 10.1270/jsbbs.22017

**Published:** 2022-12-06

**Authors:** Nobuyuki Mizuno, Hitoshi Matsunaka, Mikiko Yanaka, Masaru Nakata, Kazuhiro Nakamura, Akiko Nakamaru, Chikako Kiribuchi-Otobe, Goro Ishikawa, Makiko Chono, Koichi Hatta, Masaya Fujita, Fuminori Kobayashi

**Affiliations:** 1 Institute of Crop Science, National Agriculture and Food Research Organization, 2-1-2 Kannondai, Tsukuba, Ibaraki 305-8518, Japan; 2 Kyusyu Okinawa Agricultural Research Center, National Agriculture and Food Research Organization, 496 Izumi, Chikugo, Fukuoka 833-0041, Japan; 3 Hokkaido Agricultural Research Center, National Agriculture and Food Research Organization, 9-4 Shinsei-minami, Memuro, Kasai, Hokkaido 082-0081, Japan; 4 Tohoku Agricultural Research Center, National Agriculture and Food Research Organization, 4 Akahira, Shimo-kuriyagawa, Morioka, Iwate 020-0198, Japan

**Keywords:** heading time, genotype-environment interaction, *Ppd-1*, *Vrn-1*, wheat

## Abstract

The timing of heading is largely affected by environmental conditions. In wheat, *Vrn-1* and *Ppd-1* have been identified as the major genes involved in vernalization requirement and photoperiod sensitivity, respectively. To compare the effects of *Vrn-1* and *Ppd-1* alleles on heading time under different environments, we genotyped *Vrn-1* and *Ppd-1* homoeologues and measured the heading time at Morioka, Tsukuba and Chikugo in Japan for two growing seasons. A total of 128 Japanese and six foreign varieties, classified into four populations based on the 519 genome-wide SNPs, were used for analysis. Varieties with the spring alleles (*Vrn-D1a* or *Vrn-D1b*) at the *Vrn-D1* locus and insensitive allele (Hapl-I) at the *Ppd-D1* locus were found in earlier heading varieties. The effects of *Vrn-D1* and *Ppd-D1* on heading time were stronger than those of the other *Vrn-1* and *Ppd-1* homoeologues. Analysis of variance revealed that heading time was significantly affected by the genotype-environment interactions. Some *Vrn-1* and *Ppd-1* alleles conferred earlier or later heading in specific environments, indicating that the effect of both alleles on the timing of heading depends on the environment. Information on *Vrn-1* and *Ppd-1* alleles, together with heading time in various environments, provide useful information for wheat breeding.

## Introduction

The timing of flowering is one of the most important traits for local adaptation and is controlled by environmental cues, such as temperature and day length. Earlier flowering can escape high temperatures and drought stress during anthesis and grain filling (Bennett *et al.* 2012, Bentley *et al.* 2013). Effects of genetic factors controlling flowering time depend on plant growth conditions (environmental conditions). Understanding genotype-environment (G × E) interactions is important for wheat breeding because they often affect grain yield in wheat (Eltaher *et al.* 2021, Nehe *et al.* 2019). Thus, a study of G × E can lead to the successful evaluation of wheat varieties for stability in yield performance across environments.

Heading time, which is closely correlated with flowering time, is an important trait for breeding, since heading time can affect the yield of temperate cereals such as wheat (Mizuno *et al.* 2021). Vernalization requirement, photoperiod sensitivity, and narrow-sense earliness (earliness *per se*) are factors that determine the timing of heading in wheat. Vernalization requirement is controlled by *VERNALIZATION* genes, *Vrn-1* (Yan *et al.* 2003), *Vrn-2* (Yan *et al.* 2004a), *Vrn-3* (Yan *et al.* 2006) and *Vrn-D4* (Kippes *et al.* 2015). *Vrn-1* encodes an *APETALA1/FRUITFULL*-like (*AP1/FUL*-like) MADS-box transcription factor (Yan *et al.* 2003). One or more dominant alleles at *Vrn-1* homoeoloci confer a spring growth habit (vernalization insensitive), whereas plants without any spring-type alleles at *Vrn-1* homoeoloci show a winter growth habit (vernalization sensitive). The insertions and deletions within the promoter or a deletion within intron 1 were causal mutations of dominant alleles at the *Vrn-A1* locus (Chen *et al.* 2013, Fu *et al.* 2005, Yan *et al.* 2004b). On the one hand, the spring type of *Vrn-B1* and *Vrn-D1* alleles has been mostly due to the insertion and deletion in the intron 1 region (Fu *et al.* 2005, Yan *et al.* 2004b, Zhang *et al.* 2012). Natural variations in *VRN3*, the wheat ortholog of the *Arabidopsis FT* (*FLOWERING LOCUS T*), have been found in the B genome (Chen *et al.* 2013, Yan *et al.* 2006). The dominant allele, which conferred a spring habitat, contains a 5.3 kb insertion of a retrotransposon in the promoter region. On the other hand, the combination of mutations in all three *VRN-2* homoeologues that conferred spring growth habit has not been observed in the examined varieties of common wheat (Kippes *et al.* 2016). Photoperiod sensitivity is controlled by the *Photoperiod-1* (*Ppd-1*) gene, which encodes a protein with a sequence similarity to *Arabidopsis* *PSEUDO RESPONSE REGULATOR* 7 (*PRR7*) (Beales *et al.* 2007, Turner *et al.* 2005). Photoperiod-insensitive alleles of *Ppd-1* (*Ppd-1a*) have been identified for each homoeologues on chromosomes 2A, 2B and 2D of common wheat (Beales *et al.* 2007, Shaw *et al.* 2012, Wilhelm *et al.* 2009). Deletions or insertions in the promoter regions of *Ppd-A1*, *Ppd-B1* and *Ppd-D1* are associated with photoperiod-insensitivity in wheat (Beales *et al.* 2007, Nishida *et al.* 2013, Wilhelm *et al.* 2009). *WPCL1*, a clock gene homologue of *Arabidopsis thaliana LUX ARRHYTHMO (LUX)/PHYTOCLOCK 1* (*PCL1*), was identified as a gene conferring an early heading phenotype and controlling the expression pattern and levels of *Ppd-1* genes (Mizuno *et al.* 2012, 2016). In addition to these allelic variations in *Vrn-1* and *Ppd-1* homoeologues, copy number variations (CNVs) have also been observed in *Ppd-B1* and *Vrn-A1* (Díaz *et al.* 2012). Furthermore, many molecular genetic studies have extended our understanding of the wheat flowering regulatory network (Shi *et al.* 2019).

In barley, *Vrn-H1* and *Ppd-H1* were identified as the major stabilizing genes of the heading response for regional adaptation (Sato *et al.* 2020). *Vrn-1* and *Ppd-1* genes have been frequently identified as the major quantitative trait loci for heading time in wheat (Guedira *et al.* 2016, Iehisa *et al.* 2014, Mizuno *et al.* 2021, Nguyen *et al.* 2013). The allelic diversity of *Vrn-1* and *Ppd-1*, in addition to heading time, has been well investigated in wheat (Chen *et al.* 2018, Seki *et al.* 2011, 2013, Zhang *et al.* 2015). Recent sequencing-based studies of *Ppd-1* genes have also updated the allelic diversity (Chen *et al.* 2018). However, it remains largely unknown how the alleles of these genes affect heading time in different environments such as years or locations. To understand G × E on timing of heading in wheat, it is important to investigate the effect of *Vrn-1* and *Ppd-1* alleles in different environments. The aim of this study is to analyze the effect of the interaction between *Vrn-1* and *Ppd-1* alleles and the environment on the timing of heading and to uncover the effects of temperature on the timing of heading. In this study, we selected 134 representative wheat accessions including modern varieties and materials for breeding research in Japan and investigated the heading time of these varieties at three locations in Japan during two growing seasons and discuss the relationship between the environments and haplotypes of the *Vrn-1* and *Ppd-1* homoeologues.

## Materials and Methods

### Plant materials

A total of 134 wheat varieties were selected from recent breeding varieties, important genetic resources, promising lines, and materials for basic research based on their geographical and genealogical information. The variety names and breeding areas of the 134 varieties are listed in Supplemental Table 1.

### Measurement of heading time

These varieties were grown in fields at Morioka, Tsukuba and Chikugo in Japan during the 2019–2020 and 2020–2021 growing seasons. The sowing date for each location and season and location information is summarized in Supplemental Table 2. Each experimental plot consisted of a single 80-cm-long row with an 8 cm space between each plant. Ten individuals were grown per replicate, and measurements were taken for two replicates. The heading date was recorded when the tip of the first spike emerged from the flag leaf sheath in half of the plants for each variety. Days to heading (DH) was defined as the days from sowing to heading.

### Genotyping by amplicon sequencing

Genome-wide genotyping via amplicon sequencing was performed following the protocol described by Ishikawa *et al.* (2018). Low-quality reads and adaptors were trimmed using Trimmomatic (v0.36) with the options “SLIDINGWINDOW:4:25” and “MINLEN:40” (Bolger *et al.* 2014). The trimmed paired-end reads were mapped using BWA-mem (v0.7.15) with -L 10 and -B 10 options (Li and Durbin 2009). SNPs were detected using HaplotypeCaller in GATK 3.7 (DePristo *et al.* 2011). The minimum read count was set to 5. Heterozygous site (H) was called when two alleles had more than 40% of the total reads each.

### Construction of phylogenetic tree and structure analysis

A maximum likelihood (ML) tree was constructed using RAxML-NG (Kozlov *et al.* 2019) with the “GTR+G+ASC_LEWIS” model and bs-trees option of 1000. The best ML tree was rooted using midpoint rooting and visualized with FigTree 1.4.4 (http://tree.bio.ed.ac.uk/software/figtree/). ADMIXTURE v1.3.0 (Alexander *et al.* 2009) was used to investigate the population structure of the 134 varieties. The runs of ADMIXTURE analysis were visualized using pong v1.5 (Behr *et al.* 2016). For each value of *K*, ten ADMIXTURE analysis runs were performed with different random seeds. The best run was selected according to the highest log-likelihood value. A principal component analysis (PCA) based on covariance was performed using Tassel 5 (Bradbury *et al.* 2007).

### Genotyping of the *Vrn-1* and *Ppd-1* homoeoloci

Total DNA was extracted from leaves using a DNeasy Plant Maxi Kit (Qiagen, Germany) following the manufacturer’s protocol. We used polymerase chain reaction (PCR) primers (Supplemental Table 3) that had been shown to identify the alleles of *Vrn-1* and *Ppd-1* homoeologues in previous studies, and we amplified DNA by PCR using a T100 thermal cycler (Bio-Rad Laboratories Inc., Hercules, CA, USA) and GoTaq DNA polymerase (Promega Corp., Madison, WI, USA). The PCR conditions were as follows: denaturation at 95°C for 1 min, followed by 35 cycles of the denaturation at 95°C for 30 s, annealing for 30 s, extension at 72°C for 30 s, and then final extension at 72°C for 5 min. Information on the primer sets used in this study is presented in Supplemental Table 3. The amplicons were separated on a 2.0% agarose gel and visualized using SYBR Safe DNA Gel Stain (Invitrogen, Carlsbad, CA, USA), or separated by a capillary electrophoresis system (LabChip GX Touch HT, PerkinElmer, Inc., Waltham, MA, USA) with a DNA5K/RNA/CZE chip (PerkinElmer). Allele names of the *Vrn-1* and *Ppd-1* genes were determined according to Chen *et al.* (2018).

### Statistical analysis

Data for the two-seasons (2019–2020 and 2020–2021) from the three locations (Morioka, Tsukuba, and Chikugo) were summarized and statistically analyzed for combined analysis of variance (ANOVA) and additive main effect and multiplicative interaction (AMMI) analysis using the AMMI function in the agricolae package in R software (de Mendiburu and Yaseen 2020). Scatter plots were generated using the ggplot2 package in R software and regression lines were added with the “lm” method (Wickham 2016). Statistically significant differences between groups were determined using two-way ANOVA and Tukey-Kramer’s HSD test in R software (*P* < 0.05). Meteorological data in Morioka, Tsukuba and Kurume near Chikugo were obtained from the Japan Meteorological Agency (https://www.data.jma.go.jp).

## Results

### Evaluation of genetic diversity in wheat collection

**Fig. 1.**
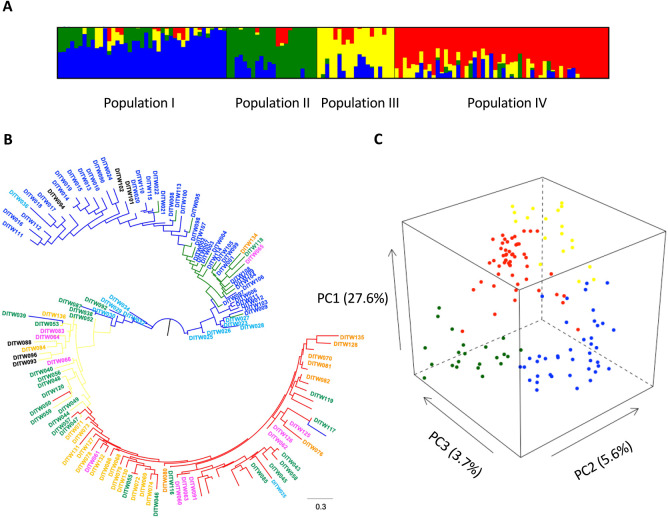
Phylogenetic tree, principal component analysis (PCA), and STRUCTURE analysis of 134 wheat accessions based on 519 genome-wide SNPs. (A) Population structure of 134 common wheat varieties inferred by ADMIXTURE (*K* = 4). Ancestry proportions for individuals were estimated using 519 SNPs. Color codes (blue, green, yellow, and red) of bars indicate typical genotypes of the inferred subpopulations. (B) A maximum likelihood tree based on 519 SNPs. IDs of varieties was color-coded by breeding area as shown in Supplemental Table 1. Blue, green, yellow, and red branches indicate Population I, II, III, and IV, respectively. (C) Principal-component analysis (PCA) of 134 varieties of common wheat based on 519 SNPs. Graph of the first three axes (PC1, PC2, and PC3) from PCA. The proportion of variance explained by each component is given in a parentheses along each axis. Blue, green, yellow, and red indicate the varieties belonging to Population I, II, III, and IV.

To evaluate the genome-wide diversity of the 134 wheat varieties, genotyping was performed using amplicon sequencing. A total of 519 SNPs were detected (minor allele frequency (MAF) >0.05 and proportion of missing data <0.2). To investigate the population structure of the 134 wheat varieties, we conducted multiple analyses (ML phylogenetic, PCA and population structure) using these 519 SNPs. Cross-validation error by ADMIXTURE analysis decreased up to *K* = 4 and *K* = 5, and then gradually increased as *K* increased (Supplemental Fig. 1A). There was one type dividing the population at *K* = 4, whereas there were three types of dividing the population out of ten in the ADMIXTURE analysis at *K* = 5 (Supplemental Fig. 1B). Thus, these results indicate that the number of clusters at *K* = 4 was suitable for the 134 wheat varieties (Fig. 1A). The PCA and ML tree also indicated that clustering agreed with that obtained by the ADMIXTURE analysis, although the varieties from Tohoku and spring varieties from Hokkaido clustered differently in the ML tree (Fig. 1B, 1C). At *K* = 4, the 134 wheat varieties were divided into four populations: spring wheat varieties from Hokkaido and the varieties from Tohoku/Hokuriku areas (Population I), winter wheat varieties from Hokkaido (Population II), varieties from Kanto/Tosan/Tokai areas (Population III), and varieties from Kinki/Chugoku/Shikoku and Kyushu areas (Population IV) (Supplemental Table 4). The foreign varieties belonged to Population I and III.

### Distribution of *Vrn-1* and *Ppd-1* alleles in wheat varieties

To examine the allele frequency of *Vrn-1* and *Ppd-1* homoeologues in the 134 varieties, we performed PCR analysis using the primer sets reported in previous studies (Chen *et al.* 2018). For *Vrn-A1* and *Vrn-B1*, the *Vrn-A1a* and *Vrn-B1a* alleles (spring type) were observed in 15 and 11 varieties, respectively, which belonged to Population I and IV (Table 1). Another spring *Vrn-A1b* allele was found in one variety, ‘Fielder’ (DITW094), belonging to Population I. For *Vrn-D1*, 63 varieties (47.0%) had the spring *Vrn-D1a* allele, and the proportion of the *Vrn-D1a* allele in Population III and IV was much higher than that in Population I and II. Another spring *Vrn-D1b* allele was observed in nine varieties, among which eight belonged to Population III and IV. For *Ppd-A1*, 24 varieties had the *Ppd-A1a* allele (insensitive allele), which was mainly found in Population I and II. The proportion of the *Ppd-A1a* allele was much higher (81.8%) in Population II than in the other populations (less than 12.2%). For *Ppd-B1*, the *Ppd-B1a* allele (insensitive allele) was found in two varieties, ‘Fukuwase komugi’ (DITW064) and ‘Abukumawase’ (DITW074), belonging to Population III and IV, respectively. Additionally, we found three CNVs of *Ppd-B1*, which were detected in bread wheat (Díaz *et al.* 2012). Hapl-II of *Ppd-B1* was mainly found in Population I and II, whereas Hapl-V of *Ppd-B1* was mainly observed in Population III and IV. For *Ppd-D1*, the majority of varieties belonging to Population III (17/19 varieties, 89.5%) and Population IV (51/52 varieties, 98.1%) had the Hapl-I of *Ppd-D1* (insensitive allele). The varieties belonging to Population I also possessed a much higher proportion of the Hapl-I of *Ppd-D1* (27/47 varieties, 65.9%) than that of the varieties belonging to Population II (5/22 varieties, 22.7%). The Hapl-III of *Ppd-D1*, which conferred late heading, was observed only in four varieties belonging to Population I [‘Hokuei’ (DITW006), ‘Akasabishirazu 1’ (DITW011), ‘Sapporo Harukomugi’ (DITW013) and ‘Harumakikomugi Norin 75’ (DITW014)]. When compared by breeding area, the allele frequencies of *Vrn-1* and *Ppd-1* differed largely between the northeastern area (Hokkaido and Tohoku/Hokuriku) and the southwestern area (Kanto/Tosan/Tokai, Kinki/Chugoku/Shikoku and Kyusyu) (Supplemental Table 4). All of varieties from Tohoku/Hokuriku and Hokkaido except for the spring-sowing varieties had no spring allele of *Vrn-1* homoeologues, whereas most varieties from southwestern area possessed one or more spring allele of *Vrn-1* homoeologues spring allele at *Vrn-1* homoeoloci. The majority of Japanese varieties had insensitive alleles at *Ppd-1* homoeoloci, whereas the spring-sowing varieties from Hokkaido rarely had them.

In total, 34 allele combinations of *Vrn-1* and *Ppd-1* homoeologues were observed in the 134 varieties (Supplemental Table 5). The frequency of allele combinations varied among the four populations, although they were similar between Population III and IV. The varieties belonging to Population III and IV mainly had *vrn-A1*/*vrn-B1*/*Vrn-D1a*/*Ppd-A1b*/Hapl-I of *Ppd-B1*/Hapl-I of *Ppd-D1* or *vrn-A1*/*vrn-B1*/*Vrn-D1a*/*Ppd-A1b*/Hapl-V of *Ppd-B1*/Hapl-I of *Ppd-D1*. The higher frequency of the spring allele of *Vrn-D1* (*Vrn-D1a* and *Vrn-D1b*) and insensitive allele of *Ppd-D1* (Hapl-I) was one of the characteristics in Population III and IV. The varieties belonging to Population I and II had the highest frequency of *vrn-A1*/*vrn-B1*/*vrn-D1*/*Ppd-A1a*/Hapl-I of *Ppd-B1*/Hapl-II of *Ppd-D1* and *vrn-A1*/*vrn-B1*/*vrn-D1*/*Ppd-A1b*/Hapl-I or Hapl-II of *Ppd-B1*/Hapl-I of *Ppd-D1* allele combinations, respectively.

### Evaluation of heading time

**Fig. 2.**
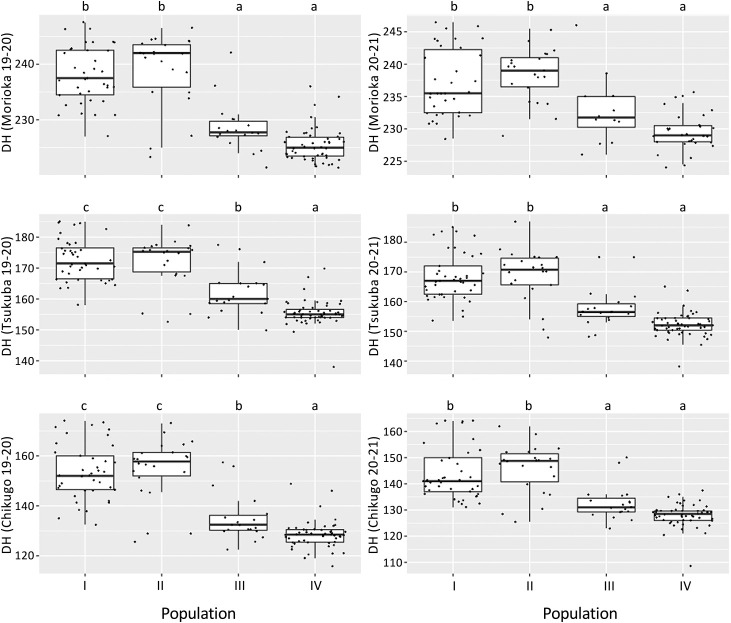
Comparison of days to heading (DH) among four populations in three locations for two seasons. The top marks the 75% quantile, and the bottom, the 25% quantile. The median (50% quantile) is marked with a thick horizontal line. The lines that protrude from the box (the whiskers) respectively show the minimum and maximum values excluding outliers. Mean values with the same letters are not significantly different (*P* > 0.05) (Tukey-Kramer’s HSD test).

To compare the heading time in different environments, DH was surveyed at three locations in Japan (Morioka, Tsukuba and Chikugo) during the 2019–2020 and 2020–2021 seasons (Supplemental Table 1). Seven varieties died in the 2019–2020 season, whereas 37 varieties died in the 2020–2021 seasons at Morioka. No variety died in Tsukuba and Chikugo during the two growing seasons. The DH of all varieties was in the order of Chikugo, Tsukuba, and Morioka. The mean DH in Tsukuba and Chikugo in the 2020–2021 season (160.7 and 136.4) was smaller than that in the 2019–2020 season (164.4 and 141.3), whereas the mean DH in Morioka in the 2019–2020 season (231.9) was smaller than that in the 2020–2021 season (234.5). The correlation coefficients between the two seasons at the three locations were high, with correlation coefficients of 0.897 at Morioka, 0.970 at Tsukuba, and 0.957 at Chikugo (Table 2). For both seasons, a strong positive correlation was also found between the locations (*R* > 0.91 and *P* < 0.001). There were significant differences in DH depending on the locations, growing seasons, and populations (Fig. 2). The varieties belonging to Population III and IV headed significantly earlier than those belonging to Population I and II. In the 2019–2020 season, significant differences in DH between Population III and IV were observed in Tsukuba and Chikugo.

### Effects of *Vrn-1* and *Ppd-1* alleles and their allele combinations on heading time

We then compared the DH among the varieties with different alleles of *Vrn-1* and *Ppd-1* homoeologues. Of the three *Vrn-1* homoeologues, a significant difference in DH consistently at three locations was observed only among the alleles of *Vrn-D1* (Supplemental Fig. 2). The spring alleles (*Vrn-D1a* or *Vrn-D1b*) at the *Vrn-D1* locus conferred significantly earlier heading than the winter alleles (*vrn-D1*). There were significant differences in DH among the alleles of the three *Ppd-1* homoeologues. The insensitive alleles of *Ppd-B1* (*Ppd-B1a* and Hapl-V) and *Ppd-D1* (Hapl-I) conferred significantly earlier heading than the sensitive alleles of *Ppd-B1* (Hapl-I and Hapl-II) and *Ppd-D1* (Hapl-II and Hapl-III). In contrast, the varieties with the sensitive allele of *Ppd-A1* (*Ppd-A1b)* headed significantly earlier than those with the insensitive allele of *Ppd-A1* (*Ppd-A1a*) (Supplemental Fig. 3). There were little significant differences in DH among the genotypes of *Vrn-A1* and *Vrn-B1* (Supplemental Figs. 2, 3). However, we confirmed *Vrn-A1a*, *Vrn-B1a* and *Ppd-A1a* conferred early heading by taking the genotypes of the other loci into consideration (Supplemental Table 5).

To confirm the effect of the allele combination of *Vrn-1* and *Ppd-1*, we first performed a two-way ANOVA. Significant interactions between vernalization requirement and photoperiod sensitivity, which were determined by the genotypes of *Vrn-1* and *Ppd-1*, were observed at Morioka and Tsukuba in the 2020–2021 season (Supplemental Table 6). Since many allele combinations had only a few varieties or one variety, it was difficult to compare them. Therefore, we focused on the allele combinations of *Vrn-D1* and *Ppd-B1*, where the number of varieties is large. Among the varieties with *Vrn-D1a*, the varieties with Hapl-I of *Ppd-B1* showed slightly earlier heading in all environments than those with Hapl-V of *Ppd-B1* (Supplemental Fig. 5). Of the six environments, the varieties with Hapl-I of *Ppd-B1* headed significantly earlier than those with Hapl-V of *Ppd-B1* only at Tsukuba for the 2019–2020 season (*P* < 0.05, independent samples *t*-test). On the contrary, among the varieties with *Vrn-D1b* or *vrn-D1*, the varieties with Hapl-V of *Ppd-B1* headed earlier than those with *Ppd-B1*.

### Effects of *Vrn-1* and *Ppd-1* haplotypes on heading time in different environment

**Fig. 3.**
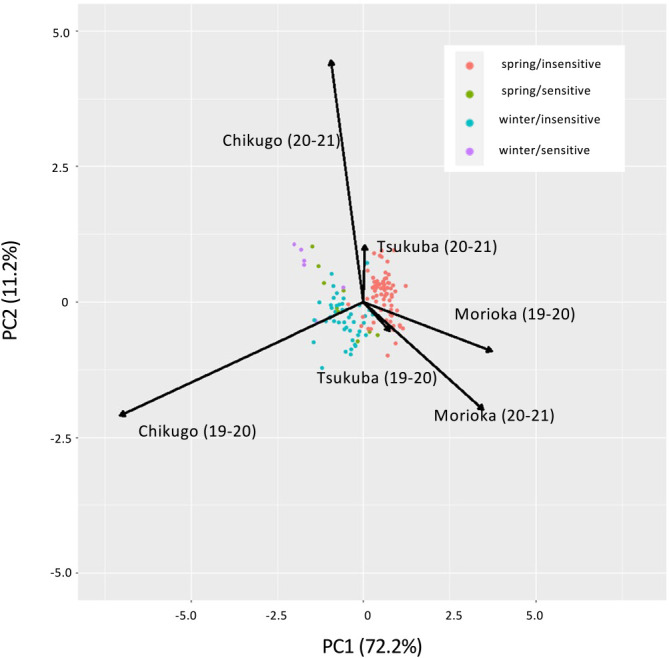
AMMI2-biplot, based on days to heading, of the 134 wheat varieties in six environments (two growing seasons at three locations), showing the effects of primary and secondary components (IPCA1 and IPCA2, respectively). IPCA1 and IPCA2 accounted for 72.2% and 11.2%, respectively, of the total genotype by environment interaction sum of squares. “spring” and “winter” indicate varieties with one or more spring allele of *Vrn-1* and without spring allele of *Vrn-1*, respectively. “insensitive” and “sensitive” indicate varieties with one or more insensitive allele of *Ppd-1* and without insensitive allele of *Ppd-1*, respectively.

**Fig. 4.**
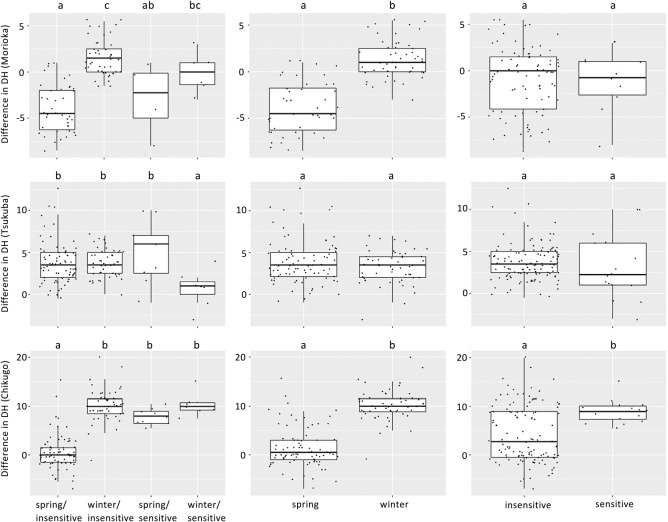
Relationship between inter-seasonal difference in DH and vernalization requirement and photoperiod sensitivity based on the allele of *Vrn-1* and *Ppd-1*. The y-axis indicates the DH for 2019–2020 season minus DH for 2020–2021 season. “spring” and “winter” indicate varieties with one or more spring allele of *Vrn-1* and without spring allele of *Vrn-1*, respectively. “insensitive” and “sensitive” indicate varieties with one or more allele of *Ppd-1* and without allele of *Ppd-1*, respectively. Top and bottom are 75% and 25% quartiles, respectively. Thick horizontal line, median (50% quartile). Whiskers represent minimum and maximum values excluding outliers. Mean values with same letters do not significantly differ (*P* > 0.05, Tukey-Kramer’s HSD test).

To investigate whether the effects of *Vrn-1* and *Ppd-1* haplotypes were altered by different environments, we compared DH between two seasons and among three locations by focusing on the haplotypes of *Vrn-1* and *Ppd-1* homoeologues. The ANOVA of the AMMI model revealed that DH was significantly (*P* < 0.01) affected by environment, genotype, and genotype-environment interaction (Table 3). Environments, genotypes and G × E interactions explained 93.2%, 6.0% and 0.7% of the total sum of squares, respectively. The first two interaction principal component analyses (IPCA1 and IPCA2) explained 72.2% and 11.2% of the G × E interaction variation, respectively. The AMMI2 biplot (Fig. 3) indicates that the 134 varieties were clearly divided into clusters according to the combination of differences in vernalization requirement and photoperiod sensitivity, which were determined by the alleles of *Vrn-1* and *Ppd-1* homoeologues. In particular, the varieties showing spring/insensitive and winter/insensitive phenotypes were clearly divided by IPCA1. In Morioka, the varieties with spring allele of *Vrn-1* tended to head earlier in the 2020–2021 season than in the 2019–2020 season, compared to the varieties without spring alleles of *Vrn-1* (Fig. 4). In contrast, the difference in the alleles of *Ppd-1* homoeologues did not significantly affect the difference in DH between the two seasons. In Chikugo, the varieties without spring allele of *Vrn-1* and insensitive allele of *Ppd-1* tended to head later in the 2019–2020 season than in the 2019–2020 season compared with the varieties with spring allele of *Vrn-1* and insensitive allele of *Ppd-1* (Fig. 4). The varieties with spring allele of *Vrn-1* and insensitive allele of *Ppd-1* showed less DH differences between the two growing seasons compared with the other varieties. Unlike Morioka and Chikugo, the differences in DH between the two growing seasons in varieties without spring allele of *Vrn-1* and insensitive allele of *Ppd-1* were less than those in the other varieties in Tsukuba. The *Vrn-D1* alleles, rather than the allele combination of *Vrn-1* and *Ppd-1* homoeologues, had a large effect on the inter-seasonal differences in DH in Morioka and Chikugo (Supplemental Fig. 5). There were little clear differences among the other allele combinations, although there were combinations with only a few varieties (data not shown).

**Fig. 5.**
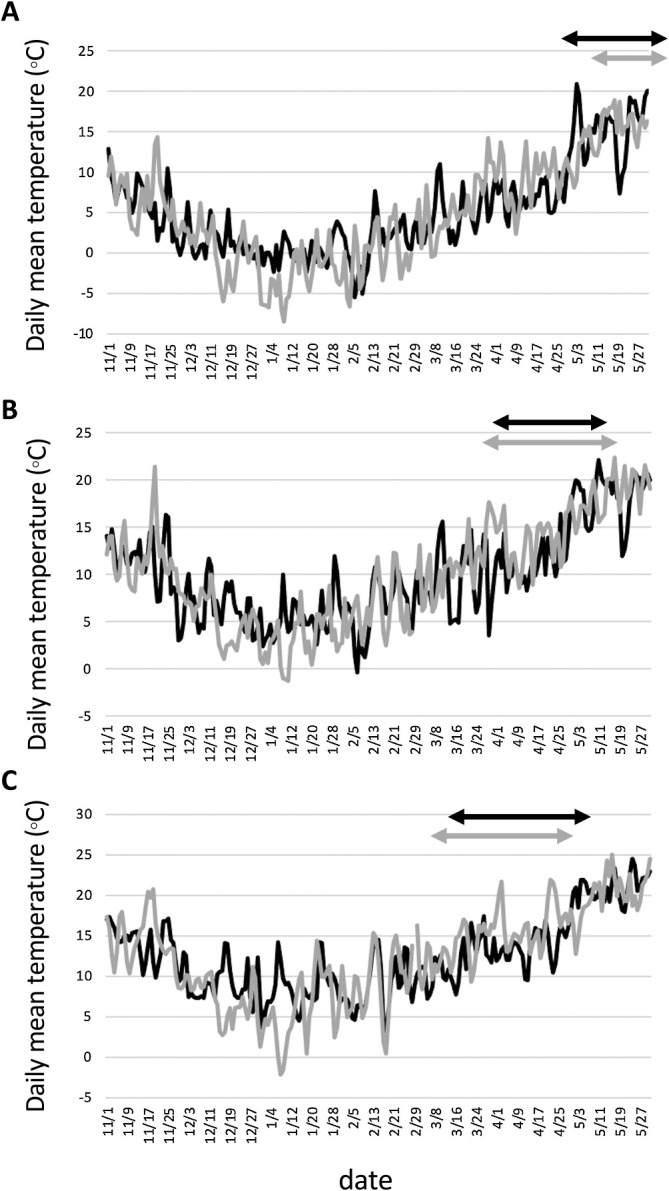
Comparison of daily mean temperature between 2019–2020 and 2020–2021 seasons in three locations. Data in Morioka (A), Tsukuba (B), and Kurume near Chikugo (C) were obtained from the Japan Meteorological Agency. Black and gray lines indicate the daily mean temperature in the 2019–2020 and 2020–2021 growing seasons, respectively. The two-headed arrows indicate days of heading in the 2019–2020 (black) and 2020–2021 (gray) growing seasons.

To study whether differences in DH between environments were dependent on haplotypes of *Vrn-1* and *Ppd-1* homoeologues, we compared the heading time between growing seasons in each location and between locations for each growing season by focusing on the haplotypes of *Vrn-1* and *Ppd-1* homoeologues. The haplotypes that showed a difference in DH between the two seasons differed at the three locations (Supplemental Figs. 4–6). In Morioka and Chikugo, DH differences between the two growing seasons differed significantly among the alleles of *Vrn-D1* and *Ppd-D1*. In Tsukuba, on the contrary, DH differences between the two growing seasons showed no significant differences among the alleles of *Vrn-D1* and *Ppd-D1*. Comparing DH among the three locations for each growing season, some alleles of *Vrn-1* and *Ppd-1* homoeologues showed different DH trends from the other alleles (Supplemental Figs. 7, 8). For example, the varieties with Hapl-III of *Ppd-D1* headed earlier in Morioka in the 2019–2020 season compared to Tsukuba and Chikugo in the 2019–2020 season. There were clear differences in the daily mean temperature during cultivation between the two seasons at the three locations (Fig. 5, Supplemental Fig. 9). All three locations experienced colder temperatures in December and January of the 2019–2020 season, but warmer temperatures in March and April of the 2020–2021 season. In Morioka, the number of days below 0°C during the 2019–2020 and 2020–2021 seasons was 32 and 55 days, respectively. Furthermore, the total amount of snowfall from December to February during the 2019–2020 and 2020–2021 seasons was 107 cm and 173 cm, respectively. The average monthly temperatures from December to April during the 2019–2020 and 2020–2021 seasons were 1.3/0.2/1.0/4.8/7.3°C and 0.2/–2.8/–0.2/5.7/9.3°C in Morioka, 6.7/5.4/6.3/9.4/11.5°C and 5.2/3.1/6.3/11.1/13.5°C in Tsukuba and 9.1/8.5/8.7/11.8/13.8°C and 7.1/6.1/9.3/13.4/16.6°C in Chikugo, respectively (Supplemental Fig. 9).

## Discussion

Environments can largely affect the timing of heading, and the timing of heading is considered an important trait for local adaptation. In wheat, *Vrn-1* and *Ppd-1* have been identified as the major genes for vernalization requirement and photoperiod sensitivity, respectively. In this study, we investigated whether the effect of *Vrn-1* and *Ppd-1* haplotypes on the timing of heading depends on the environment using 134 varieties, including 128 Japanese and six foreign varieties. Based on the genome-wide 519 SNPs, the ADMIXTURE analysis indicated that the 134 varieties were largely divided into four populations, named Population I, II, III and IV (Fig. 1A). The ML tree and PCA plot also supported this classification (Fig. 1B, 1C). Most varieties from the northeastern region (Hokkaido and Tohoku) were included in Population I and II. On the one hand, most of the varieties from the southwestern region (from Kanto to Kyushu) belonged to Population III and IV. The foreign varieties from the USA and Hungary, ‘Fielder’ (DITW094) and ‘GK Szemes’ (DITW101), belonged to Population I. On the other hand, the varieties from China, ‘Sumai 3’ (DITW088) and ‘Chinese Spring’ (DITW096), and Italy, ‘Ardito’ (DITW093), belonged to Population III. Many genetic resources, including foreign varieties (mainly from North America and Europe), have been introduced during breeding in northern areas of Japan, whereas limited genetic resources have been used in southwestern Japan. The clustering in this study reflected the history of wheat breeding in Japan, in agreement with a study by Kobayashi *et al.* (2016), which investigated the genetic diversity of Japanese wheat core collection based on genome-wide SNPs using genotyping-by-sequencing (GBS). Compared to the study by Kobayashi *et al.* (2016), this study included more modern varieties, and the phylogenetic relationship reflects recent breeding processes. For example, three varieties, ‘Biwahonami’ (DITW065), ‘Kanto 143’ (DITW118) and ‘Hakei W 1320’ (DITW134), from southwestern Japan, belonged to Population II (Fig. 1A, Supplemental Table 1), indicating that these varieties possess the genetic background of the winter wheat variety in Hokkaido. The winter wheat variety ‘Kitahonami’ (DITW001), belonging to Population II, is a leading variety in Hokkaido and is used as a breeding material because of its desirable traits such as yield and quality (Yanagisawa *et al.* 2007). Actually, ‘Kitahonami’ is used in the pedigree of these varieties.

We then examined the allele frequency of *Vrn-1* and *Ppd-1* homoeologues and heading time at three locations for two growing seasons using these 134 varieties. The varieties belonging to Population III and IV headed significantly earlier than those belonging to Population I and II (Fig. 2). The varieties belonging to Population III and IV frequently had *Vrn-D1a*/*Vrn-D1b* and Hapl-I of *Ppd-D1*, whereas the frequencies of these alleles were low in Population I and II (Table 1). Since either *Vrn-D1a*/*Vrn-D1b* or Hapl-I of *Ppd-D1* did not confer early heading (Supplemental Table 5, Supplemental Fig. 5), both *Vrn-D1a*/*Vrn-D1b* and Hapl-I of *Ppd-D1* were essential for early heading. In the west of Kanto, early maturing wheat varieties must avoid preharvest sprouting and Fusarium head blight during the rainy season. Thus, early varieties with both *Vrn-D1a*/*Vrn-D1b* and Hapl-I of *Ppd-D1* were selected in the west of Kanto. On the other hand, the varieties from Tohoku/Hokuriku and winter varieties from Hokkaido possessed no spring alleles of *Vrn-1* (Supplemental Table 4), which showed less tolerance to large amounts of snow and low temperatures. Thus, the preferred haplotypes of *Ppd-1* and *Vrn-1* have been selected in each region of Japan to adapt to the environment and reflected well on the population structure and breeding area. For *Ppd-B1*, CNVs, as well as mutations in the promoter region, have been known to confer photoperiod insensitivity (Díaz *et al.* 2012, Nishida *et al.* 2013). *Ppd-B1a* and Hapl-V of *Ppd-B1* conferred early heading at three locations. *Ppd-B1a* is less preferred than Hapl-V even in west of the Kanto region (Supplemental Table 4). This is possibly because the varieties with *Ppd-B1a* started floral development and stem elongation earlier than the other insensitive alleles of *Ppd-1* (Tanio and Kato 2007). By taking the genotypes of the other loci into consideration, it was shown that *Ppd-A1a*, *Vrn-A1a* and *Vrn-B1a* also contributed to early heading (Supplemental Table 5). Since the number of these varieties is small, we need to analyze on a larger scale to confirm the detailed effect of these genotypes on timing of heading. There were large DH variations even within the varieties with same haplotype of *Vrn-1* and *Ppd-1* (Supplemental Fig. 5, Supplemental Table 5), indicating the other gene or genes that determined differences in timing of heading among Japanese wheat varieties. The genotypes of *Ppd-1* in Japanese wheat varieties have been investigated (Seki *et al.* 2011, 2013), and the information in these studies have generally been used for breeding. In this study, we included recent breeding varieties and investigated the detailed alleles of each gene and their effects on DH in Japanese wheat varieties. This data provides more practical haplotype information for breeding, which allows us to adjust the timing of heading.

Genotype-environment (G × E) interactions affect the timing of heading in durum wheat (Mohammadi *et al.* 2011). In this study, we also showed that DH was significantly (*P* < 0.01) affected by the genotype-environment interaction (Table 3). To investigate the interaction between haplotypes and environments on heading time, we compared the DH between environments by focusing on the haplotypes of *Vrn-1* and *Ppd-1* homoeologues. The AMMI2 biplot (Fig. 3) showed that there were 134 varieties that were clearly divided into clusters according to the varieties showing different vernalization requirements and photoperiod sensitivity, which were determined by the haplotypes of *Vrn-1* and *Ppd-1* homoeologues. In Morioka, the varieties with spring allele of *Vrn-D1* headed earlier in the 2019–2020 season than in the 2020–2021 season compared with the varieties without spring alleles of *Vrn-1* (Supplemental Fig. 4). In contrast, the difference in photoperiod sensitivity, which was determined by the alleles of *Ppd-1* homoeologues, had little effect on the DH differences between the two growing seasons. Comparing temperature between the two seasons, the 2019–2020 season had higher temperatures during the winter months (December and January) and fewer days with daily mean temperatures below 0°C (Fig. 5). It has long been known that there is a correlation between growth habit and freezing tolerance and that wheat genotypes with a spring growth habit are less freezing tolerant than genotypes with a winter growth habit (Hayes and Aamodt 1927). However, more varieties died in the 2020–2021 season than in the 2019–2020 season due to snow mold rather than freezing. In fact, during the 2019–2020 season, the total amount of snowfall from December to February was 1.62 times higher than that during the 2020–2021 season. All of these varieties had one or more spring alleles of *Vrn-1* (Supplemental Table 1). Spring varieties probably delayed heading owing to damage by snow mold. Thus, the DH difference between the two growing seasons in Morioka could be explained by the snow mold tolerance associated with the vernalization requirement. Recent genetic studies have shown that snow mold resistance is closely associated with the *Vrn-1* homoeoloci on chromosomes 5A and 5D (Lozada *et al.* 2019, Nishio *et al.* 2020). The *Vrn-A1b* and *Vrn-D1b* alleles were less susceptible to environmental factors than the *Vrn-A1a* and *Vrn-D1a* alleles in Morioka. No varieties with *Vrn-A1b* and *Vrn-D1b* died in the two growing seasons in Morioka (Supplemental Table 1). This suggests that varieties with the *Vrn-A1b* and *Vrn-D1b* alleles were more resistant to snow mold than those with the *Vrn-A1a* and *Vrn-D1a* alleles. In Tsukuba and Chikugo, unlike Morioka, heading was basically earlier in the 2020–2021 season than the 2019–2020 season (Fig. 4), although the 2019–2020 season had higher effective accumulated temperatures until mid-April than the 2020–2021 season. The earlier heading in the 2020–2021 season might be due to higher temperatures from March to April in the 2020–2021 season than the 2019–2020 season. The timing of the temperature increase and the range of the temperature change might explain the different effects of the *Vrn-1* and *Ppd-1* alleles on the seasonal DH difference among the three locations. In Chikugo, the spring varieties with reduced photoperiod sensitivity (especially with *Vrn-D1a/Vrn-D1b* and Hapl-I of *Ppd-D1*) headed earlier in the 2019–2020 season than in the 2020–2021 season, whereas there were no significant inter-seasonal differences in DH between the varieties with different vernalization requirement and between the varieties with different photoperiod sensitivity in Tsukuba (Fig. 4). There is little difference in the timing of the day length change from short day to long day between Tsukuba and Chikugo, and the temperatures in Chikugo are on an average 2°C to 3°C higher than those in Tsukuba (Fig. 5, Supplemental Fig. 9). The latitude of Tsukuba and Chikugo are 36.03 and 33.21, respectively (Supplemental Table 2). Although the difference in day length during winter is about 15 minutes at maximum between Tsukuba and Chikugo, the date when the day changes from short day to long day is the same around March 17 in Tsukuba and Chikugo. Thus, the difference in temperature rather than the difference in day length between Tsukuba and Chikugo might explain the differences in responsiveness to winter warming in the spring and insensitive varieties between Tsukuba and Chikugo. Further analysis is needed to understand the difference between Tsukuba and Chikugo using growth chambers with fine temperature control. The long-term accumulation of data for heading time at various locations, together with meteorological data, will make it possible to predict the timing of heading and to breed wheat varieties that head at the appropriate time, even in fluctuating environments.

## Author Contribution Statement

HM, MY, MN, KN, AN, KH, CK and MF conducted the field evaluations. GI performed amplicon sequencing. MC and FK performed genotyping using the PCR markers. HM, MF, KH, MK, MC and FK selected wheat materials for this study and designed the experimental studies in the field. NM analyzed and interpreted the raw data. NM also conceived the draft of the manuscript and wrote the manuscript. FK conceived and designed the study for raw data acquisition and managed all the raw data. FK also revised the manuscript accordingly and provided final confirmation. FK was responsible for all of materials used in this study and of raw data for heading time and genotypes of these materials. NM was responsible for the analysis of all raw data and its interpretation. As FK and NM have equal and separate responsibilities for this manuscript, they are the corresponding authors. All authors have read and approved the final manuscript.

## Supplementary Material

Supplemental Figures

Supplemental Tables

## Figures and Tables

**Table 1.** Allele frequency of *Vrn-1* and *Ppd-1* homoeologues and their mean days to heading in the 134 wheat varieties

Gene	Allele	Population I	Population II	Population III	Population IV	Total	Morioka (19–20)	Tsukuba (19–20)	Chikugo (19–20)	Morioka (20–21)	Tsukuba (20–21)	Chikugo (20–21)
*Vrn-A1*	***Vrn-A1a***	11	0	0	4	15	235.0	170.5	146.6	240.3	165.5	141.5
	*Vrn-A1b*	1	0	0	0	1	242.5	174.5	144.0	241.5	164.0	138.0
	*vrn-A1*	29	22	19	48	118	231.5	163.5	140.6	234.1	160.1	135.8
*Vrn-B1*	***Vrn-B1a***	9	0	0	2	11	237.8	172.3	147.9	241.7	166.9	141.5
	*vrn-B1*	25	22	19	49	115	231.3	163.4	140.2	234.1	159.9	135.8
	*^a^*NA	7	0	0	1	8	234.7	167.6	147.6	236.9	163.6	139.2
*Vrn-D1*	***Vrn-D1a***	5	3	14	41	63	226.4	157.5	130.1	230.9	153.6	129.4
	***Vrn-D1b***	1	0	2	6	9	227.1	156.1	131.1	228.2	153.8	128.6
	*vrn-D1*	35	19	3	5	62	238.2	172.5	154.2	237.5	168.8	144.8
*Ppd-A1*	***Ppd-A1a***	5	18	0	1	24	238.1	170.5	151.9	237.3	166.5	142.9
	*Ppd-A1b*	36	4	19	51	110	230.5	163.0	139.0	233.7	159.4	135.0
*Ppd-B1*	***Ppd-B1a***	0	0	1	1	2	221.5	149.8	120.8	226.0	146.8	121.8
	Hapl-I	25	16	9	31	81	232.8	165.2	143.3	235.5	162.1	138.4
	Hapl-II	13	5	0	1	19	236.1	170.2	149.1	235.9	165.6	140.3
	**Hapl-V**	3	1	9	19	32	228.0	159.7	132.8	231.2	155.3	130.2
*Ppd-D1*	**Hapl-I**	27	5	17	51	100	229.2	160.5	135.3	232.2	156.8	131.9
	Hapl-II	10	17	2	1	30	240.5	174.9	157.5	239.8	171.3	148.3
	Hapl-III	4	0	0	0	4	242.3	182.1	168.6	243.7	180.1	160.3

*^a^* NA: Not available. The spring alleles of *Vrn-1* and insensitive allele of *Ppd-1* are shown in bold.

**Table 2.** Correlation coefficients of heading time among the six environments

	Tsukuba (19–20)	Chikugo (19–20)	Morioka (20–21)	Tsukuba (20–21)	Chikugo (20–21)
Morioka (19–20)	0.952*	0.948*	0.897*	0.942*	0.908*
Tsukuba (19–20)		0.958*	0.924*	0.970*	0.945*
Chikugo (19–20)			0.881*	0.971*	0.957*
Morioka (20–21)				0.925*	0.919*
Tsukuba (20–21)					0.973*

* *P* < 0.001.

**Table 3.** Additive main effect and multiplicative interaction (AMMI) analysis of variance across six environments

	Degree of freedom	Sum of square	Mean sum of square	F value	% of total sum of square	% of G × E
Genotype	133	148135.0	1114.0	474.2*	6.0	
Environment	5	2287198.0	457440.0	7514.3*	93.2	
Replication	6	365.0	61.0	25.9*	0.02	
Interaction	627	18044.0	29.0	12.3*	0.7	
Residuals	725	1703.0	2.0			
IPCA1	137	11906.1	86.9	37.0*		72.2
IPCA2	135	1841.7	13.6	5.8*		11.2
IPCA3	133	1403.7	10.6	4.5*		8.5
IPCA4	131	849.3	6.5	2.8*		5.2
IPCA5	129	487.7	3.8	1.6*		3

* *P* < 0.001.
