# Supplementary material for: Allelic variations of Vrn-1 and Ppd-1 genes in Japanese wheat varieties reveal the genotype-environment interaction for heading time
Source: Breed Sci. 2022 Dec 6;72(5):343–54. doi: 10.1270/jsbbs.22017 (PMC9895800; doi:10.1270/jsbbs.22017)
Supplement: Supplementary file 1 — Supplemental Figures [file 72_343_s1.pdf]

Mizuno et al./Breeding Science
doi: 10.1270/jsbbs.22017

A

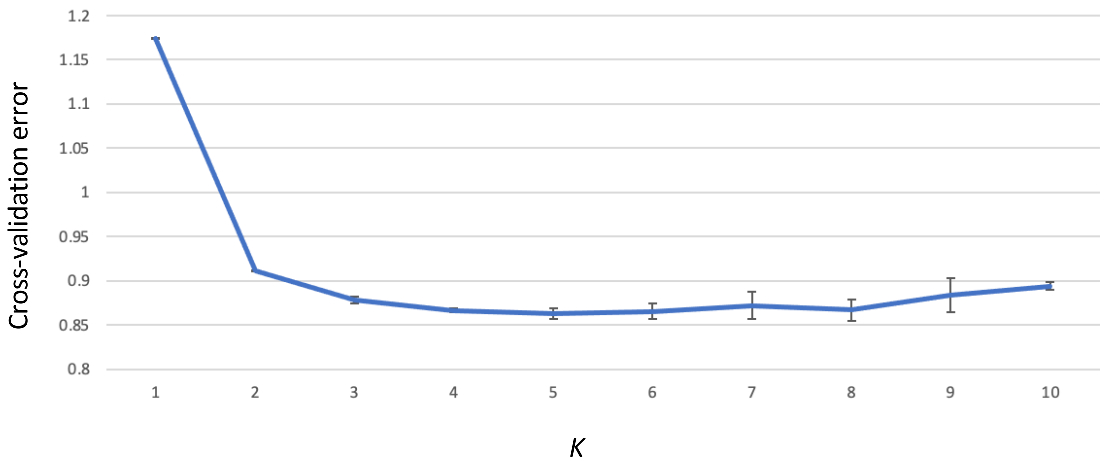

B

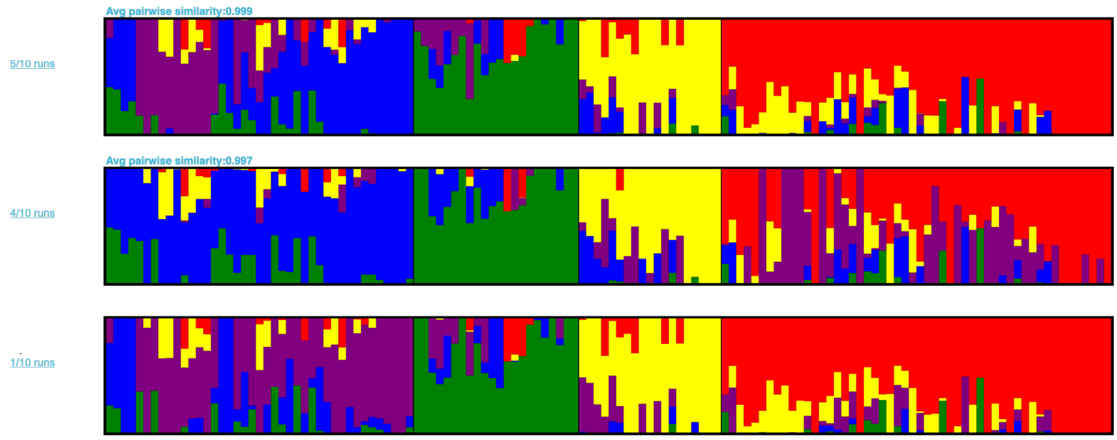

Supplemental Fig. 1. Population differentiation of the 134 wheat varieties. (A) Cross-validation errors of ancestral population assignment for different numbers of clusters by ADMIXTURE (K = 1–10). Mean cross-validation errors by 10 ADMIXTURE runs are shown with standard deviations. (B) Population structure of 134 wheat varieties inferred using ADMIXTURE (*K* = 5). Ten runs of ADMIXTURE were visualized using pong v1.5 (Behr *et al.* 2016).

.

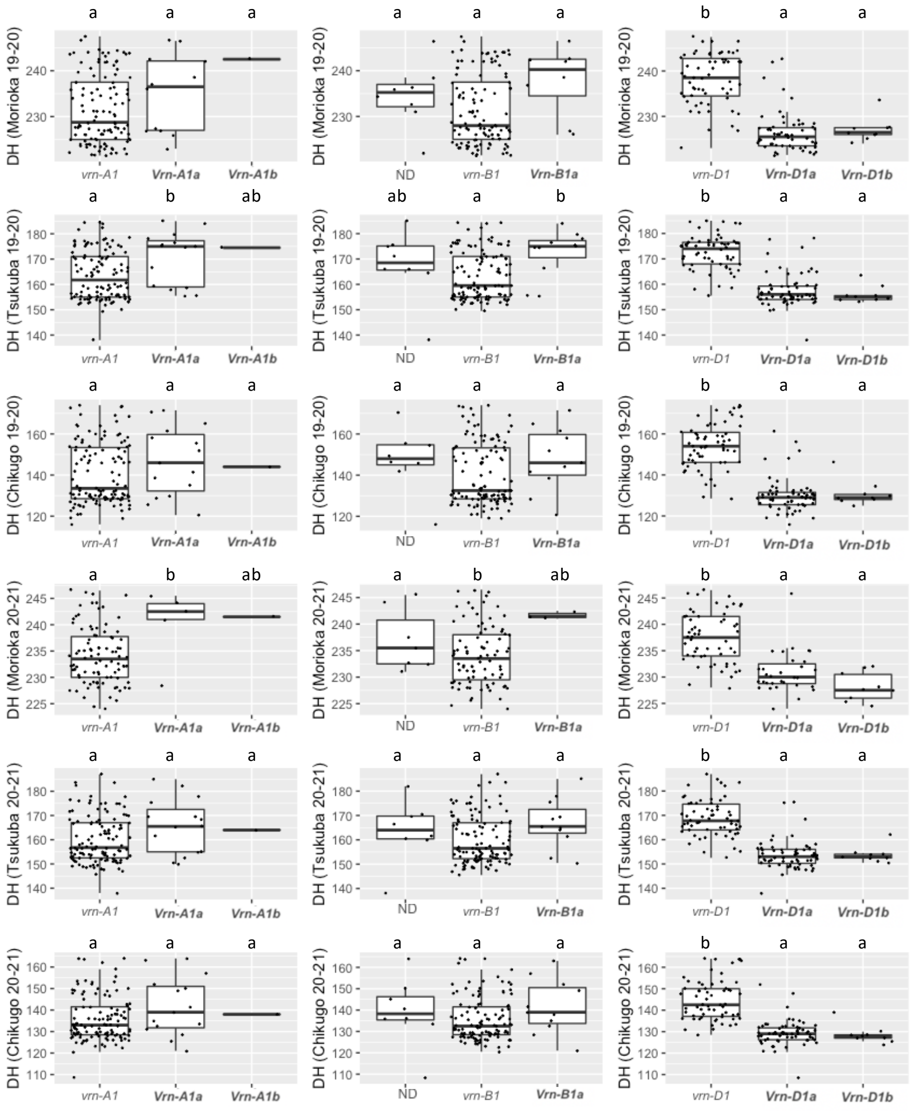

Supplemental Fig. 2. Comparison of days to heading (DH) among alleles of *Vrn-1* homoeologues in three locations for two seasons. The top marks the 75% quantile, and the bottom, the 25% quantile. The median (50% quantile) is marked with a thick horizontal line. The lines that protrude from the box (the whiskers) respectively show the minimum and maximum values excluding outliers. Mean values with the same letters are not significantly different ($P > 0.05$) (Tukey-Kramer's HSD test). The spring alleles of *Vrn-1* and insensitive allele of *Ppd-1* are shown in bold.

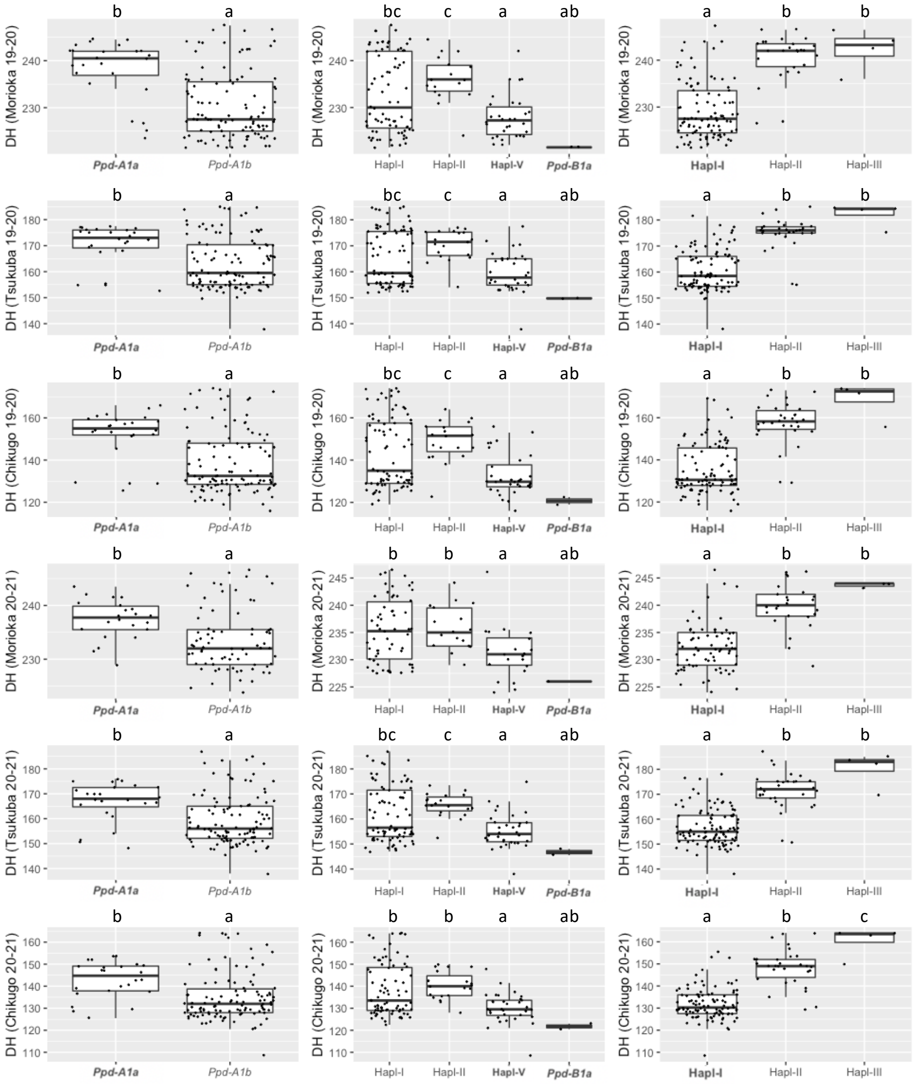

Supplemental Fig. 3. Comparison of days to heading (DH) among alleles of *Ppd-1* homoeologues in three locations for two seasons. The top marks the 75% quantile, and the bottom, the 25% quantile. The median (50% quantile) is marked with a thick horizontal line. The lines that protrude from the box (the whiskers) respectively show the minimum and maximum values excluding outliers. Mean values with the same letters are not significantly different ($P > 0.05$) (Tukey-Kramer's HSD test). The spring alleles of *Vrn-1* and insensitive allele of *Ppd-1* are shown in bold.

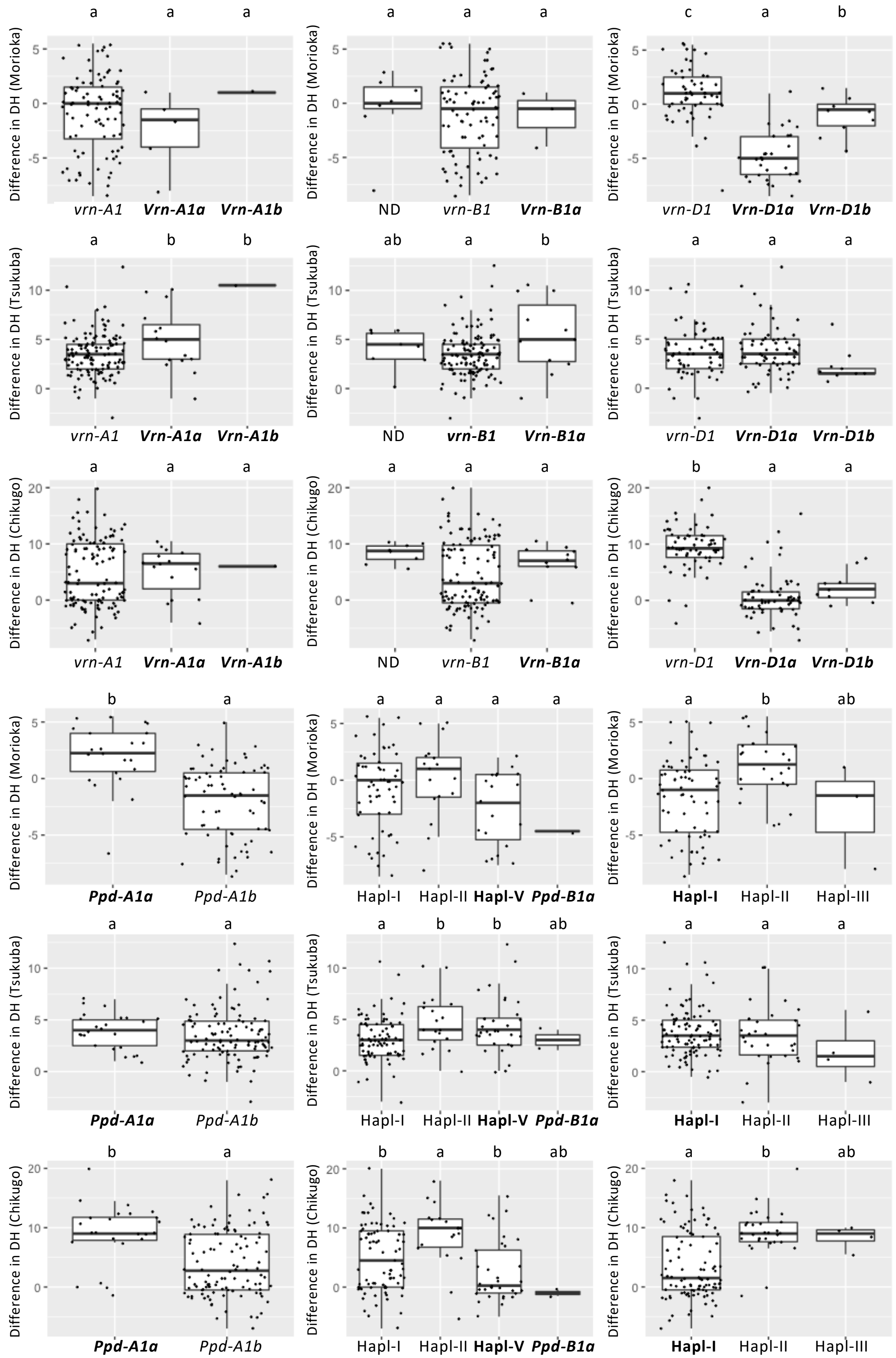

Supplemental Fig. 4. Comparison of seasonal DH differences among haplotypes of *Vrn-1* and *Ppd-1* homoeologues. The y-axis indicates DH for 2019-2020 season minus DH for 2020-2021 season. The top marks the 75% quantile, and the bottom, the 25% quantile. The median (50% quantile) is marked with a thick horizontal line. The lines that protrude from the box (the whiskers) respectively show the minimum and maximum values excluding outliers. Mean values with the same letters are not significantly different ($P > 0.05$) (Tukey-Kramer's HSD test). The spring alleles of *Vrn-1* and insensitive allele of *Ppd-1* are shown in bold.

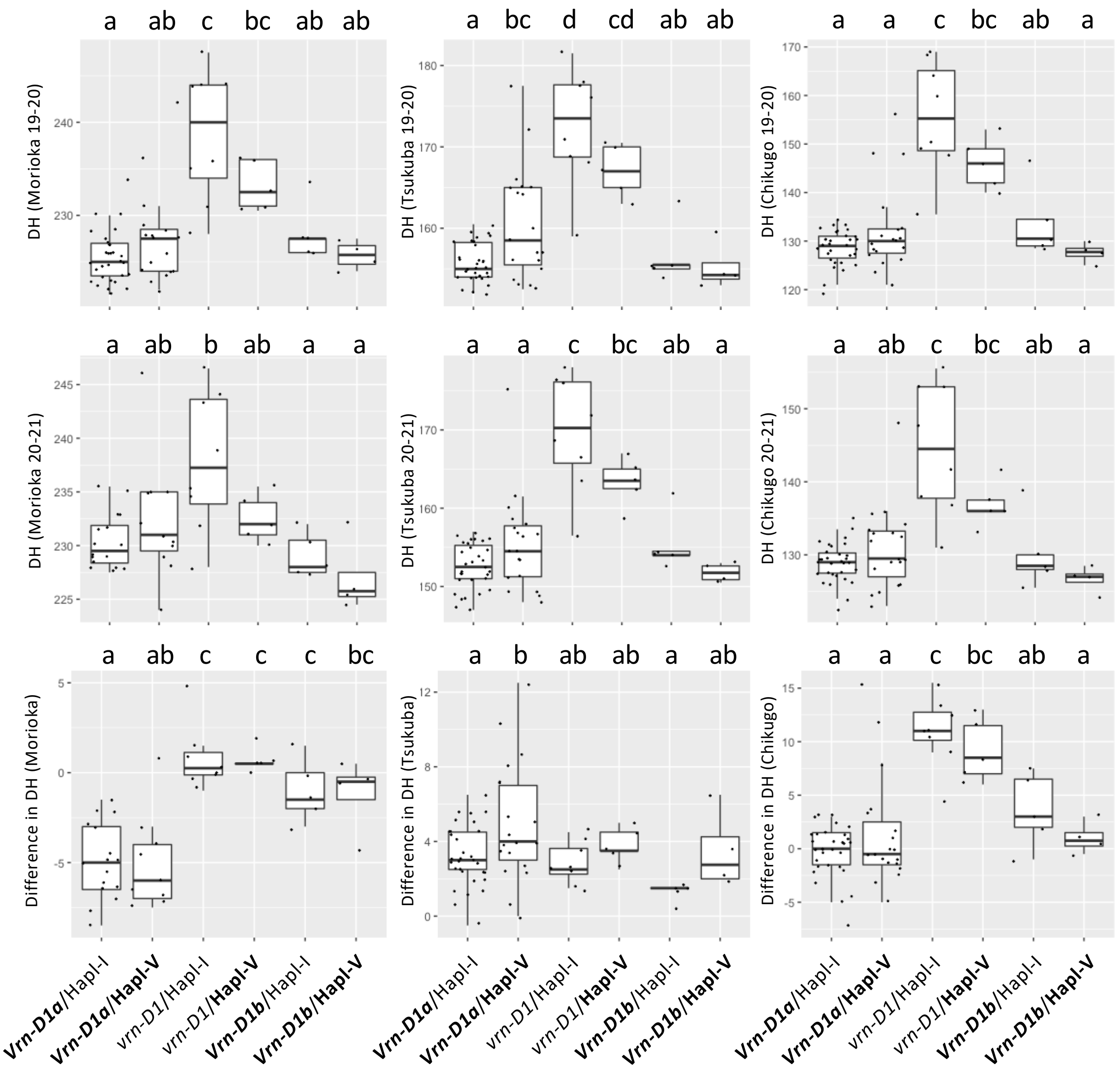

Supplemental Fig. 5. Effect of allele combination of *Vrn-D1* and *Ppd-B1* on heading time and inter-seasonal differences in DH among the varieties with *vrn-A1*/*vrn-B1*/*Ppd-A1b*/Hapl-I (*Ppd-D1*). The y-axis indicates DH for 2019–2020 season minus DH for 2020–2021 season. The top marks the 75% quantile, and the bottom, the 25% quantile. The median (50% quantile) is marked with a thick horizontal line. The lines that protrude from the box (the whiskers) respectively show the minimum and maximum values excluding outliers. Mean values with the same letters are not significantly different ($P > 0.05$) (Tukey-Kramer's HSD test). The spring alleles of *Vrn-D1* and insensitive allele of *Ppd-B1* are shown in bold.

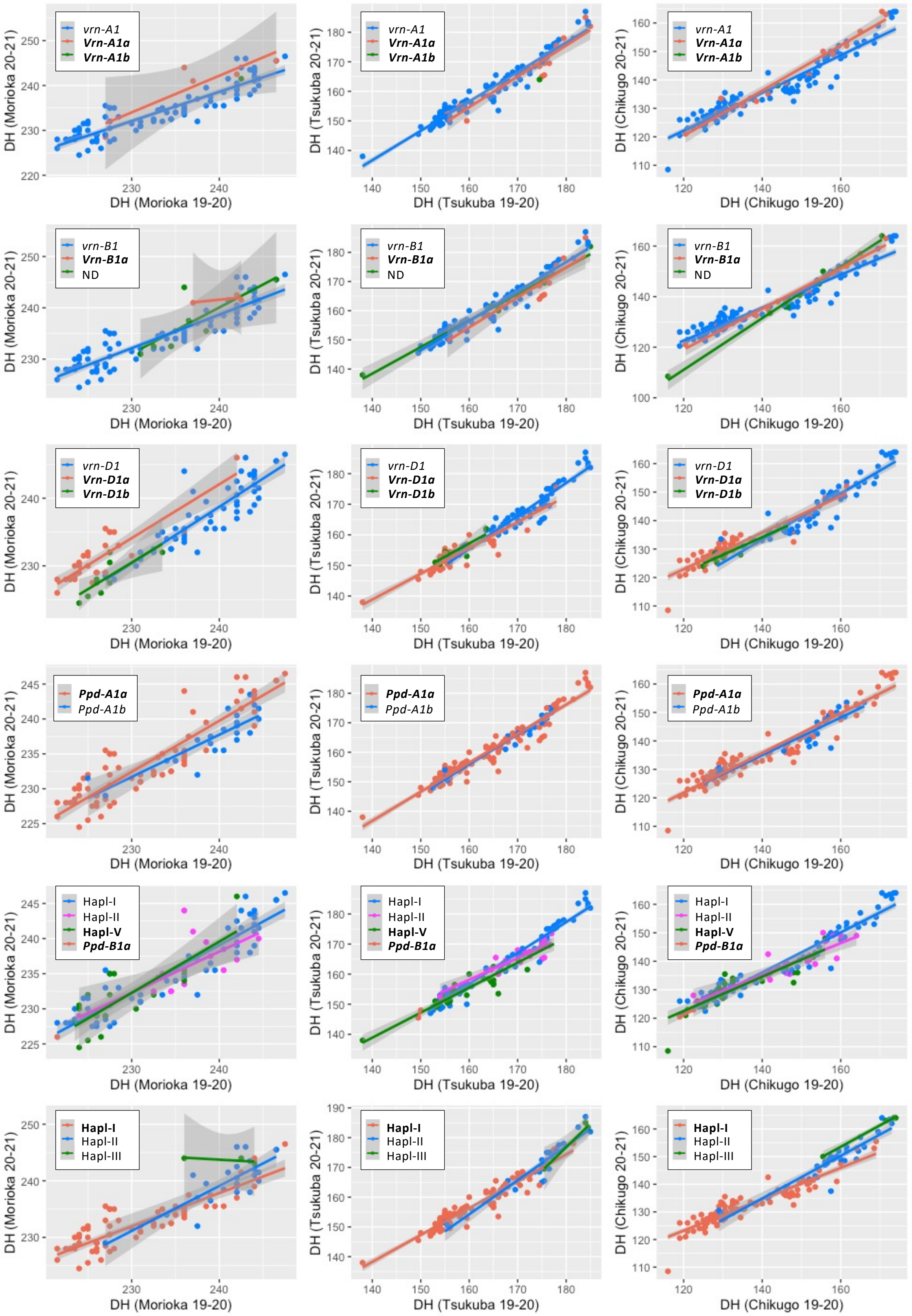

Supplemental Fig. 6. Comparison of heading time between two seasons in three locations. Cultivars are color-coded based on alleles of each *Vrn-1* and *Ppd-1* homoeologues. The spring alleles of *Vrn-1* and insensitive allele of *Ppd-1* are shown in bold.

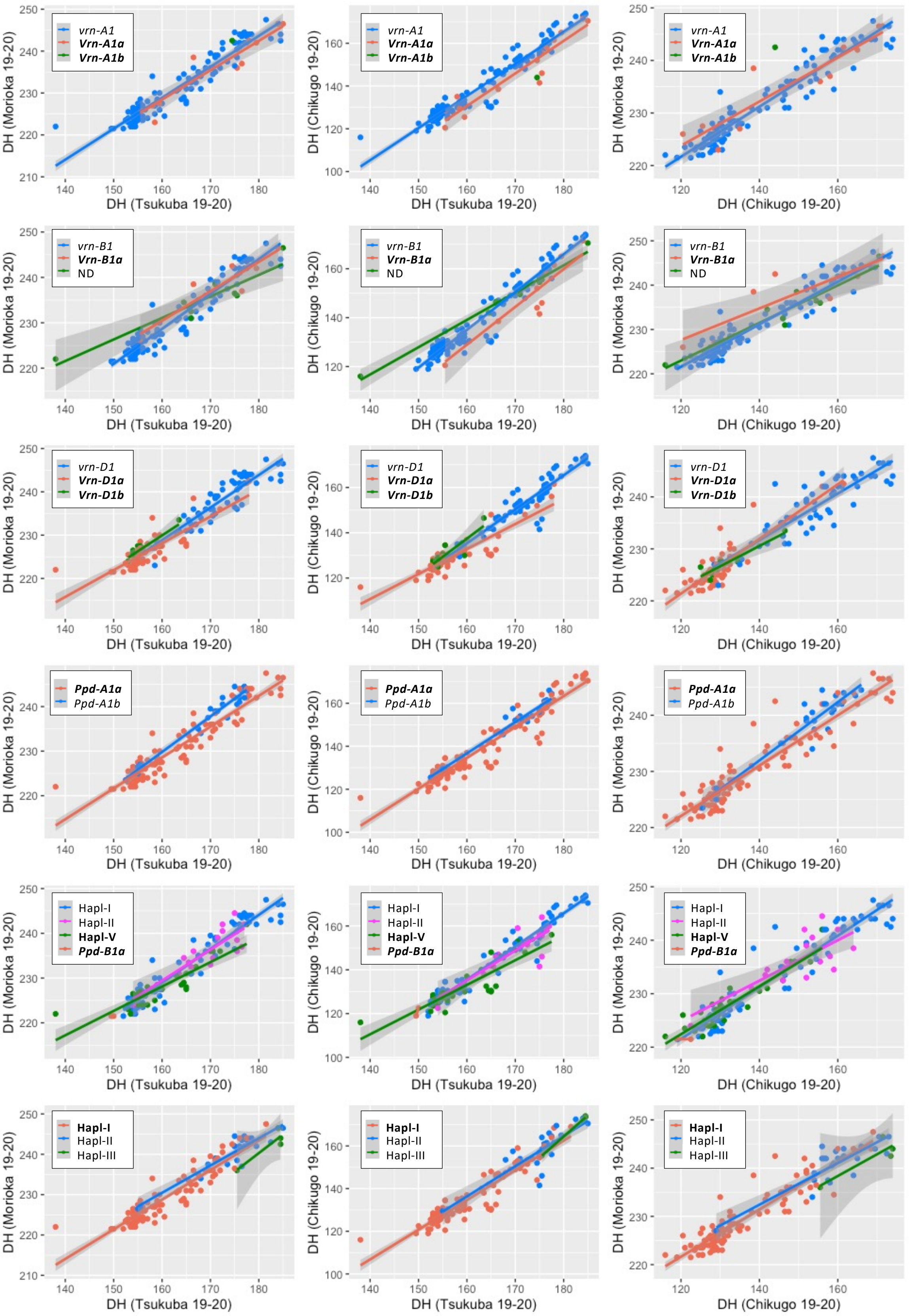

Supplemental Fig. 7. Comparison of days to heading (DH) among three locations in the 2019-2020 season. Varieties are color-coded based on alleles of each *Vrn-1* and *Ppd-1* homoeologues. The spring alleles of *Vrn-1* and insensitive allele of *Ppd-1* are shown in bold.

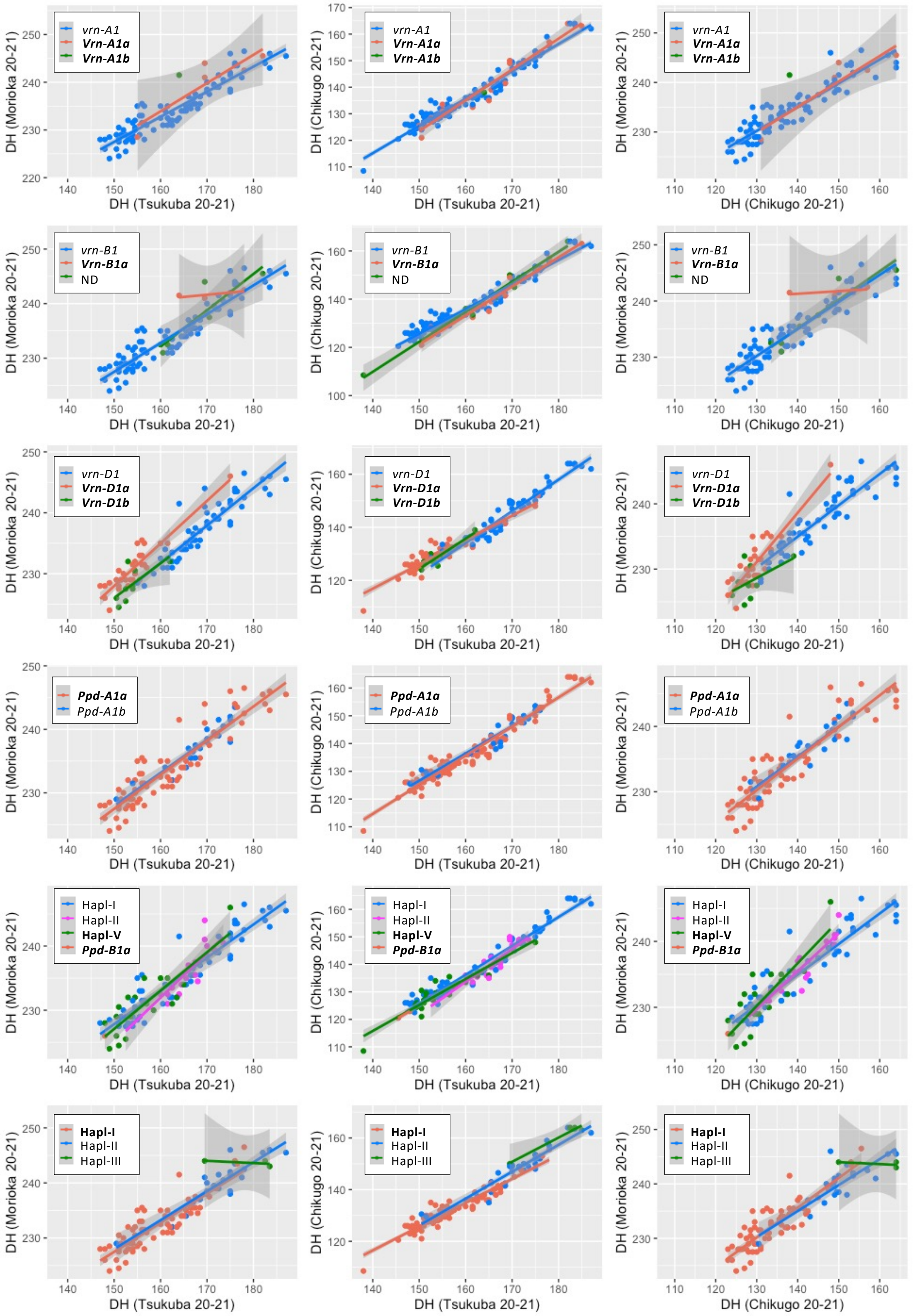

Supplemental Fig. 8. Comparison of days to heading (DH) among three locations in the 2020-2021 season. Varieties are color-coded based on alleles of each *Vrn-1* and *Ppd-1* homoeologues. The spring alleles of *Vrn-1* and insensitive allele of *Ppd-1* are shown in bold.

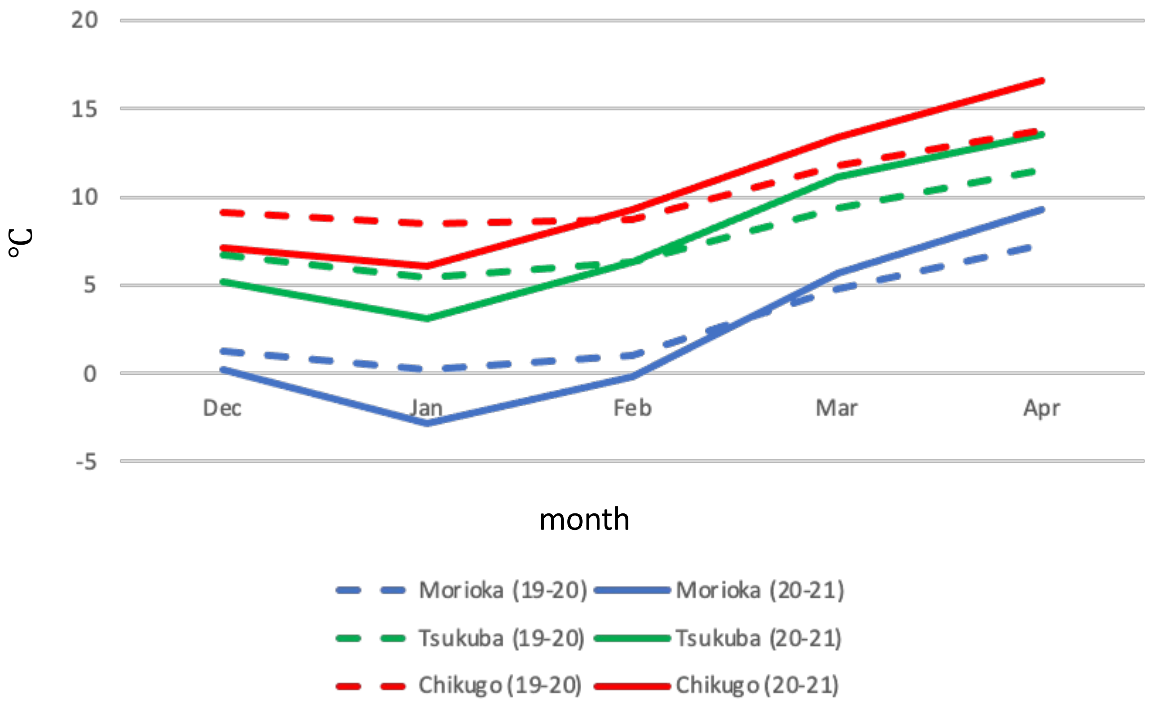

Supplemental Fig. 9. Comparison of monthly mean temperature between 2019-2020 and 2020-2021 seasons at three locations. Data in Morioka, Tsukuba and Kurume near Chikugo were obtained from the Japan Meteorological Agency.
