# Supplementary material for: Allelic variations of Vrn-1 and Ppd-1 genes in Japanese wheat varieties reveal the genotype-environment interaction for heading time
Source: Breed Sci. 2022 Dec 6;72(5):343–54. doi: 10.1270/jsbbs.22017 (PMC9895800; doi:10.1270/jsbbs.22017)
Supplement: Supplementary file 2 — Supplemental Tables [file 72_343_s2.pdf]

Mizuno et al./Breeding Science
doi: 10.1270/jsbbs.22017

Supplemental Table 1. List of varieties used in this study

ID	Variety name	Breeding area	Population	*Vrn-A1*	*Vrn-B1*	*Vrn-D1*	*Ppd-A1*	*Ppd-B1*	*Ppd-D1*	Vernalization requirement	Photoperid sensitivity	Morioka (19-20)	Tsukuba (19-20)	Chikugo (19-20)	Morioka (20-21)	Tsukuba (20-21)	Chikugo (20-21)
DITW001	Kitahonami	Hokkaido	II	*vrn-A1*	*vrn-B1*	*vrn-D1*	*Ppd-A1a*	Hapl-I	Hapl-II	winter	insensitive	241	176	159	241.5	172.5	150
DITW002	Kitamoe	Hokkaido	II	*vrn-A1*	*vrn-B1*	*vrn-D1*	*Ppd-A1a*	Hapl-I	Hapl-II	winter	insensitive	244	176.5	161.5	242	175	153.5
DITW003	Hokushin	Hokkaido	II	*vrn-A1*	*vrn-B1*	*vrn-D1*	*Ppd-A1b*	Hapl-II	Hapl-II	winter	sensitive	238.5	175.5	164	239.5	171.5	149
DITW004	Chihoku komugi	Hokkaido	II	*vrn-A1*	*vrn-B1*	*vrn-D1*	*Ppd-A1a*	Hapl-I	Hapl-II	winter	insensitive	243.5	177.5	164.5	243.5	176	152
DITW005	Horoshiri komugi	Hokkaido	I	*vrn-A1*	*vrn-B1*	*vrn-D1*	*Ppd-A1b*	Hapl-I	Hapl-I	winter	insensitive	244	178	168.5	243.5	176.5	153
DITW006	Hokuei	Hokkaido	I	*vrn-A1*	*vrn-B1*	*vrn-D1*	*Ppd-A1b*	Hapl-I	Hapl-III	winter	sensitive	244	184.5	174	243	183.5	164
DITW007	Kitasachiho	Hokkaido	II	*vrn-A1*	*vrn-B1*	*vrn-D1*	*Ppd-A1b*	Hapl-I	Hapl-II	winter	sensitive	244	178.5	169.5	241	177.5	159
DITW008	Tsurukichi	Hokkaido	I	*vrn-A1*	*vrn-B1*	*vrn-D1*	*Ppd-A1b*	Hapl-I	Hapl-I	winter	insensitive	244	176	160	239	172	147.5
DITW009	Takune komugi	Hokkaido	II	*vrn-A1*	*vrn-B1*	*vrn-D1*	*Ppd-A1a*	Hapl-II	Hapl-II	winter	insensitive	242	177	160	240.5	173.5	149
DITW010	Dawson 1	[b]Hokkaido (S)	I	*Vrn-A1a*	[a]NA	*vrn-D1*	*Ppd-A1b*	Hapl-I	Hapl-II	spring	sensitive	246.5	185	170.5	245.5	182	164
DITW011	Akasabishirazu 1	Hokkaido	I	*vrn-A1*	*vrn-B1*	*vrn-D1*	*Ppd-A1b*	Hapl-I	Hapl-III	winter	sensitive	242.5	184.5	173.5	244	182.5	164
DITW012	Muka komugi	Hokkaido	I	*vrn-A1*	*vrn-B1*	*vrn-D1*	*Ppd-A1b*	Hapl-I	Hapl-II	winter	sensitive	243	182.5	172.5	246	183.5	163.5
DITW013	Sapporo Harukomugi	[b]Hokkaido (S)	I	*Vrn-A1a*	*Vrn-B1a*	*vrn-D1*	*Ppd-A1b*	Hapl-I	Hapl-III	spring	sensitive	246.5	184	171.5	[a]NA	185	163
DITW014	Harumakikomugi Norin 75	[b]Hokkaido (S)	I	*Vrn-A1a*	[a]NA	*vrn-D1*	*Ppd-A1b*	Hapl-II	Hapl-III	spring	sensitive	236	175.5	155.5	244	169.5	150
DITW015	Haruhikari	[b]Hokkaido (S)	I	*Vrn-A1a*	*Vrn-B1a*	*vrn-D1*	*Ppd-A1b*	Hapl-II	Hapl-II	spring	sensitive	237	176.5	158	241	169.5	149
DITW016	Haruyutaka	[b]Hokkaido (S)	I	*Vrn-A1a*	*Vrn-B1a*	*vrn-D1*	*Ppd-A1b*	Hapl-I	Hapl-II	spring	sensitive	242	179.5	165	242.5	178	157
DITW017	Haruhinode	[b]Hokkaido (S)	I	*Vrn-A1a*	*Vrn-B1a*	*Vrn-D1a*	*Ppd-A1b*	Hapl-I	Hapl-II	spring	sensitive	242.5	178	161.5	[a]NA	175.5	152
DITW018	Harunoakebono	[b]Hokkaido (S)	I	*Vrn-A1a*	*Vrn-B1a*	*vrn-D1*	*Ppd-A1b*	Hapl-II	Hapl-II	spring	sensitive	[a]NA	175.5	146	[a]NA	165.5	139
DITW019	Harukirari	[b]Hokkaido (S)	I	*Vrn-A1a*	*Vrn-B1a*	*vrn-D1*	*Ppd-A1b*	Hapl-II	Hapl-II	spring	sensitive	[a]NA	175	141.5	[a]NA	165	135
DITW020	Yumechikara	Hokkaido	I	*vrn-A1*	*vrn-B1*	*vrn-D1*	*Ppd-A1a*	Hapl-II	Hapl-I	winter	insensitive	240.5	172.5	153.5	235.5	167.5	139
DITW021	Kitanokaori	Hokkaido	I	*vrn-A1*	*vrn-B1*	*vrn-D1*	*Ppd-A1b*	Hapl-I	Hapl-I	winter	insensitive	247.5	181.5	169	246.5	178	155.5
DITW022	Minorinochikara	Hokkaido	II	*vrn-A1*	*vrn-B1*	*vrn-D1*	*Ppd-A1a*	Hapl-II	Hapl-I	winter	insensitive	242	172.5	151.5	237	166	140
DITW023	Nanbu komugi	Tohoku/Hokuriku	I	*vrn-A1*	[a]NA	*vrn-D1*	*Ppd-A1b*	Hapl-II	Hapl-I	winter	insensitive	234.5	164.5	142	232.5	161.5	133.5
DITW024	Kitakami komugi	Tohoku/Hokuriku	I	*vrn-A1*	*vrn-B1*	*vrn-D1*	*Ppd-A1b*	Hapl-V	Hapl-I	winter	insensitive	236	170.5	153	235.5	167	141.5
DITW025	Hachiman komugi	Tohoku/Hokuriku	I	*vrn-A1*	*vrn-B1*	*vrn-D1*	*Ppd-A1b*	Hapl-I	Hapl-I	winter	insensitive	236	169	149	234.5	166.5	138
DITW026	Norin 27	Tohoku/Hokuriku	I	*vrn-A1*	[a]NA	*vrn-D1*	*Ppd-A1b*	Hapl-I	Hapl-I	winter	insensitive	238.5	171	149.5	235.5	166.5	140.5
DITW027	Tohoku 118	Tohoku/Hokuriku	I	*vrn-A1*	*vrn-B1*	*vrn-D1*	*Ppd-A1b*	Hapl-II	Hapl-I	winter	insensitive	236	166.5	147	233.5	162.5	135.5
DITW028	Norin 33	Tohoku/Hokuriku	I	*vrn-A1*	*vrn-B1*	*vrn-D1*	*Ppd-A1b*	Hapl-II	Hapl-I	winter	insensitive	234.5	170.5	159	232.5	164	141
DITW029	Shimofusa komugi	Tohoku/Hokuriku	I	*vrn-A1*	*vrn-B1*	*vrn-D1*	*Ppd-A1b*	Hapl-I	Hapl-I	winter	insensitive	235	171	150.5	235.5	168.5	141.5
DITW030	Aoba komugi	Tohoku/Hokuriku	I	*vrn-A1*	*vrn-B1*	*vrn-D1*	*Ppd-A1b*	Hapl-II	Hapl-I	winter	insensitive	233.5	165.5	141.5	235	165.5	142.5
DITW031	Nebarigoshi	Tohoku/Hokuriku	IV	*vrn-A1*	*vrn-B1*	*vrn-D1*	*Ppd-A1b*	Hapl-V	Hapl-I	winter	insensitive	236	170	149	234	165	136
DITW032	Yukichikara	Tohoku/Hokuriku	I	*vrn-A1*	[a]NA	*vrn-D1*	*Ppd-A1b*	Hapl-II	Hapl-I	winter	insensitive	232.5	166	146	232.5	160	136
DITW033	Yukiharuka	Tohoku/Hokuriku	IV	*vrn-A1*	*vrn-B1*	*vrn-D1*	*Ppd-A1b*	Hapl-V	Hapl-I	winter	insensitive	230.5	163	140	230	158.5	133
DITW034	Natsukogane	Tohoku/Hokuriku	I	*vrn-A1*	[a]NA	*vrn-D1*	*Ppd-A1b*	Hapl-I	Hapl-I	winter	insensitive	231	166	146.5	231	160.5	136
DITW035	Mochihime	Tohoku/Hokuriku	IV	*vrn-A1*	*vrn-B1*	*vrn-D1*	*Ppd-A1b*	Hapl-V	Hapl-I	winter	insensitive	232.5	167	146	232	163.5	137.5
DITW036	Ginganochikara	Tohoku/Hokuriku	I	*vrn-A1*	*vrn-B1*	*vrn-D1*	*Ppd-A1b*	Hapl-I	Hapl-I	winter	insensitive	231	168	147.5	232	163.5	137
DITW037	Shirane komugi	Kanto/Tosan/Tokai	III	*vrn-A1*	*vrn-B1*	*vrn-D1*	*Ppd-A1b*	Hapl-V	Hapl-I	winter	insensitive	231	165	142	231	162.5	136
DITW038	Zenkoji komugi	Kanto/Tosan/Tokai	I	*vrn-A1*	*vrn-B1*	*Vrn-D1a*	*Ppd-A1b*	Hapl-V	Hapl-I	spring	insensitive	[a]NA	165	148	231	157	132.5
DITW039	Shunyou	Kanto/Tosan/Tokai	I	*vrn-A1*	*vrn-B1*	*Vrn-D1b*	*Ppd-A1b*	Hapl-I	Hapl-I	winter	insensitive	233.5	163.5	146.5	232	162	139
DITW040	Kinuhime	Kanto/Tosan/Tokai	III	*vrn-A1*	*vrn-B1*	*vrn-D1*	*Ppd-A1b*	Hapl-I	Hapl-I	winter	insensitive	228	159	135.5	228	156.5	131
DITW041	Yumekaori	Kanto/Tosan/Tokai	I	*Vrn-A1a*	*vrn-B1*	*vrn-D1*	*Ppd-A1b*	Hapl-I	Hapl-I	spring	insensitive	227	158	135	228.5	155	131
DITW042	Hanamanten	Kanto/Tosan/Tokai	IV	*vrn-A1*	*vrn-B1*	*Vrn-D1a*	*Ppd-A1b*	Hapl-V	Hapl-I	spring	insensitive	[a]NA	153	128.5	224	149	125
DITW043	Kinunonami	Kanto/Tosan/Tokai	IV	*vrn-A1*	*vrn-B1*	*Vrn-D1a*	*Ppd-A1b*	Hapl-I	Hapl-I	spring	insensitive	226	155	131	229	155.5	129.5
DITW044	Satonosora	Kanto/Tosan/Tokai	III	*vrn-A1*	*vrn-B1*	*Vrn-D1b*	*Ppd-A1b*	Hapl-I	Hapl-I	spring	insensitive	227.5	155.5	134.5	227.5	154	128
DITW045	Ayahikari	Kanto/Tosan/Tokai	IV	*vrn-A1*	*vrn-B1*	*Vrn-D1a*	*Ppd-A1b*	Hapl-I	Hapl-I	spring	insensitive	225	155	129.5	230	152	127.5
DITW046	Aobanokoi	Kanto/Tosan/Tokai	IV	*vrn-A1*	*vrn-B1*	*Vrn-D1a*	*Ppd-A1b*	Hapl-I	Hapl-I	spring	insensitive	223	154	130.5	[a]NA	152	129
DITW047	Bandowase	Kanto/Tosan/Tokai	III	*vrn-A1*	*vrn-B1*	*Vrn-D1a*	*Ppd-A1b*	Hapl-I	Hapl-I	spring	insensitive	224	154	125.5	[a]NA	148.5	126
DITW048	Tamaizumi	Kanto/Tosan/Tokai	III	*vrn-A1*	*vrn-B1*	*Vrn-D1a*	*Ppd-A1b*	Hapl-I	Hapl-I	spring	insensitive	230	158.5	132.5	[a]NA	156	129.5
DITW049	Fukuho komugi	Kanto/Tosan/Tokai	III	*vrn-A1*	*vrn-B1*	*Vrn-D1a*	*Ppd-A1b*	Hapl-I	Hapl-I	spring	insensitive	226	156	127.5	[a]NA	154.5	129
DITW050	Yumeshihou	Kanto/Tosan/Tokai	IV	*Vrn-A1a*	*Vrn-B1a*	*vrn-D1*	*Ppd-A1b*	Hapl-I	Hapl-I	spring	insensitive	227	155.5	128.5	[a]NA	152.5	128.5
DITW051	Kinuazuma	Kanto/Tosan/Tokai	IV	*vrn-A1*	*vrn-B1*	*Vrn-D1a*	*Ppd-A1b*	Hapl-V	Hapl-I	spring	insensitive	228	157	128	235	156.5	129
DITW052	Toyoho komugi	Kanto/Tosan/Tokai	III	*vrn-A1*	*vrn-B1*	*Vrn-D1a*	*Ppd-A1b*	Hapl-V	Hapl-I	spring	insensitive	227.5	160	137	235	160	133.5
DITW053	Aira komugi	Kanto/Tosan/Tokai	III	*vrn-A1*	*vrn-B1*	*Vrn-D1b*	*Ppd-A1b*	Hapl-V	Hapl-I	spring	insensitive	227.5	159.5	130	232	153	127
DITW054	Fujimi komugi	Kanto/Tosan/Tokai	IV	*vrn-A1*	*vrn-B1*	*Vrn-D1a*	*Ppd-A1b*	Hapl-V	Hapl-I	spring	insensitive	225	158.5	131	232	154.5	129.5
DITW055	Kumakirari	Kanto/Tosan/Tokai	IV	*vrn-A1*	*vrn-B1*	*Vrn-D1b*	*Ppd-A1b*	Hapl-I	Hapl-I	spring	insensitive	226	155.5	128.5	228	154	125.5
DITW056	Tamaizumi R	Kanto/Tosan/Tokai	III	*vrn-A1*	*vrn-B1*	*Vrn-D1a*	*Ppd-A1b*	Hapl-I	Hapl-I	spring	insensitive	227	158.5	131	233	155.5	129.5
DITW057	Uraramochi	Kanto/Tosan/Tokai	IV	*vrn-A1*	*vrn-B1*	*Vrn-D1a*	*Ppd-A1b*	Hapl-I	Hapl-I	spring	insensitive	226	159.5	132	[a]NA	157	131.5

DITW058	Kinuakari	Kanto/Tosan/Tokai	IV	*vrn-A1*	*vrn-B1*	*Vrn-D1a*	*Ppd-A1b*	Hapl-I	Hapl-I	spring	insensitive	223	154.5	128	228	153	127.5
DITW059	Yumeakari	Kanto/Tosan/Tokai	III	*vrn-A1*	*vrn-B1*	*Vrn-D1a*	*Ppd-A1b*	Hapl-I	Hapl-I	spring	insensitive	224.5	160.5	130.5	231.5	156	130.5
DITW060	Fukusayaka	Kinki/Chugoku/Shikoku	IV	*vrn-A1*	*vrn-B1*	*Vrn-D1a*	*Ppd-A1b*	Hapl-I	Hapl-I	spring	insensitive	223	154	128.5	[a]NA	151.5	128
DITW061	Fukuharuka	Kinki/Chugoku/Shikoku	IV	*vrn-A1*	*vrn-B1*	*Vrn-D1a*	*Ppd-A1b*	Hapl-V	Hapl-I	spring	insensitive	222	153.5	125.5	[a]NA	149.5	124.5
DITW062	Fukuhonoka	Kinki/Chugoku/Shikoku	IV	*vrn-A1*	*vrn-B1*	*Vrn-D1a*	*Ppd-A1b*	Hapl-I	Hapl-I	spring	insensitive	222	154.5	125.5	[a]NA	149	122.5
DITW063	Shirasagi komugi	Kinki/Chugoku/Shikoku	IV	*vrn-A1*	*vrn-B1*	*Vrn-D1a*	*Ppd-A1b*	Hapl-I	Hapl-I	spring	insensitive	228.5	159	133.5	233	156	133.5
DITW064	Fukuwase komugi	Kinki/Chugoku/Shikoku	III	*vrn-A1*	*vrn-B1*	*Vrn-D1a*	*Ppd-A1b*	*Ppd-B1a*	Hapl-I	spring	insensitive	221.5	150	122.5	226	148	123
DITW065	Biwahonami	Kinki/Chugoku/Shikoku	II	*vrn-A1*	*vrn-B1*	*Vrn-D1a*	*Ppd-A1a*	Hapl-I	Hapl-I	spring	insensitive	223.5	152.5	125.5	[a]NA	148	125.5
DITW066	Ushio komugi	Kinki/Chugoku/Shikoku	III	*vrn-A1*	*vrn-B1*	*Vrn-D1a*	*Ppd-A1b*	Hapl-V	Hapl-I	spring	insensitive	228.5	164	132.5	[a]NA	158.5	133
DITW068	Asakaze komugi	Kyushu	IV	*vrn-A1*	*vrn-B1*	*Vrn-D1a*	*Ppd-A1b*	Hapl-I	Hapl-I	spring	insensitive	223.5	152.5	124	228.5	149	124
DITW069	Shirogane komugi	Kyushu	IV	*vrn-A1*	*vrn-B1*	*Vrn-D1a*	*Ppd-A1b*	Hapl-I	Hapl-I	spring	insensitive	225.5	153	127	227.5	151	129
DITW070	Chikugoizumi	Kyushu	IV	*vrn-A1*	*vrn-B1*	*Vrn-D1a*	*Ppd-A1b*	Hapl-V	Hapl-I	spring	insensitive	223	153	123.5	[a]NA	149.5	126
DITW071	Iwainodaichi	Kyushu	IV	*vrn-A1*	*vrn-B1*	*Vrn-D1b*	*Ppd-A1b*	Hapl-I	Hapl-I	spring	insensitive	226	154	129	227.5	152.5	130
DITW072	Nishiharuka	Kyushu	IV	*vrn-A1*	*vrn-B1*	*Vrn-D1a*	*Ppd-A1b*	Hapl-I	Hapl-I	spring	insensitive	223.5	155	129	230	154.5	132
DITW073	Kinuiroha	Kyushu	IV	*vrn-A1*	*vrn-B1*	*Vrn-D1a*	*Ppd-A1b*	Hapl-I	Hapl-I	spring	insensitive	221.5	152	119	228	147	126
DITW074	Abukumawase	Kyushu	IV	*vrn-A1*	*vrn-B1*	*Vrn-D1a*	*Ppd-A1b*	*Ppd-B1a*	Hapl-I	spring	insensitive	221.5	149.5	119	[a]NA	145.5	120.5
DITW075	Minamino komugi	Kyushu	IV	*vrn-A1*	*vrn-B1*	*Vrn-D1a*	*Ppd-A1b*	Hapl-V	Hapl-I	spring	insensitive	224	155	127	[a]NA	151.5	128
DITW076	Minaminokaori	Kyushu	IV	*Vrn-A1a*	*vrn-B1*	*vrn-D1*	*Ppd-A1b*	Hapl-I	Hapl-I	spring	insensitive	223	158.5	129.5	[a]NA	155	133.5
DITW077	Harukazefuwari	Kyushu	IV	*vrn-A1*	*vrn-B1*	*Vrn-D1a*	*Ppd-A1b*	Hapl-I	Hapl-I	spring	insensitive	227.5	159	132	235	155	131.5
DITW078	Sachikaori	Kyushu	IV	*vrn-A1*	*vrn-B1*	*Vrn-D1a*	*Ppd-A1b*	Hapl-V	Hapl-I	spring	insensitive	224	156	126	230.5	151	126
DITW079	Gogatsu komugi	Kyushu	IV	*vrn-A1*	*vrn-B1*	*Vrn-D1a*	*Ppd-A1b*	Hapl-V	Hapl-I	spring	insensitive	226	156	129.5	229	153.5	129.5
DITW080	Chikugomaru	Kyushu	IV	*vrn-A1*	*vrn-B1*	*Vrn-D1b*	*Ppd-A1b*	Hapl-V	Hapl-I	spring	insensitive	224	153	127.5	224.5	151	127
DITW081	Towaizumi	Kyushu	IV	*vrn-A1*	*vrn-B1*	*Vrn-D1b*	*Ppd-A1b*	Hapl-V	Hapl-I	spring	insensitive	225	154.5	128	225.5	152.5	128.5
DITW082	Saikai 188	Kyushu	IV	*vrn-A1*	*vrn-B1*	*Vrn-D1a*	*Ppd-A1b*	Hapl-I	Hapl-I	spring	insensitive	222	154	124.5	[a]NA	148.5	129
DITW083	Shin Chunaga	Kinki/Chugoku/Shikoku	III	*vrn-A1*	*vrn-B1*	*Vrn-D1a*	*Ppd-A1b*	Hapl-V	Hapl-I	spring	insensitive	228	165	130	[a]NA	158	133
DITW084	Eshima shinriki	Kyushu	III	*vrn-A1*	*vrn-B1*	*Vrn-D1a*	*Ppd-A1b*	Hapl-V	Hapl-I	spring	insensitive	227.5	165	130.5	[a]NA	156.5	135.5
DITW085	Kanto 107	Kanto/Tosan/Tokai	IV	*vrn-A1*	*vrn-B1*	*Vrn-D1a*	*Ppd-A1b*	Hapl-V	Hapl-I	spring	insensitive	224	157	128.5	230	154.5	129
DITW086	Saikai 165	Kyushu	IV	*vrn-A1*	*vrn-B1*	*Vrn-D1a*	*Ppd-A1b*	Hapl-I	Hapl-I	spring	insensitive	226	156.5	128.5	229	154	130
DITW087	Igachikugo	Kanto/Tosan/Tokai	I	*vrn-A1*	*vrn-B1*	*Vrn-D1a*	*Ppd-A1b*	Hapl-V	Hapl-I	spring	insensitive	231	166	132.5	[a]NA	153.5	134
DITW088	Sumai 3	China	III	*vrn-A1*	*vrn-B1*	*Vrn-D1a*	*Ppd-A1b*	Hapl-V	Hapl-I	spring	insensitive	236	172	148	235	161.5	136
DITW089	Gokuwase 4-15	Kinki/Chugoku/Shikoku	IV	*vrn-A1*	*vrn-B1*	*Vrn-D1a*	*Ppd-A1b*	Hapl-II	Hapl-I	spring	insensitive	224	154	122.5	229	152.5	128
DITW090	Norin 10	Tohoku/Hokuriku	I	*vrn-A1*	*vrn-B1*	*vrn-D1*	*Ppd-A1b*	Hapl-II	Hapl-I	winter	insensitive	233	170	152	234.5	168	142
DITW091	Norin 26	Kinki/Chugoku/Shikoku	IV	*vrn-A1*	*vrn-B1*	*Vrn-D1a*	*Ppd-A1b*	Hapl-I	Hapl-I	spring	insensitive	227	158.5	132.5	235.5	156	132.5
DITW092	Norin 7	Kanto/Tosan/Tokai	I	*vrn-A1*	*vrn-B1*	*vrn-D1*	*Ppd-A1b*	Hapl-II	Hapl-I	winter	insensitive	231	163.5	138	231	161.5	133
DITW093	Ardito	Itary	III	*vrn-A1*	*vrn-B1*	*vrn-D1*	*Ppd-A1b*	Hapl-I	Hapl-II	winter	sensitive	[a]NA	176	157.5	238.5	175	150
DITW094	Fielder	USA	I	*Vrn-A1b*	*Vrn-B1a*	*vrn-D1*	*Ppd-A1b*	Hapl-I	Hapl-I	spring	insensitive	242.5	174.5	144	241.5	164	138
DITW095	Hokkai 240	Hokkaido	II	*vrn-A1*	*vrn-B1*	*vrn-D1*	*Ppd-A1a*	Hapl-I	Hapl-II	winter	insensitive	242	176	161	239.5	171.5	148.5
DITW096	Chinese Spring	China	III	*vrn-A1*	*vrn-B1*	*Vrn-D1a*	*Ppd-A1b*	Hapl-V	Hapl-II	spring	insensitive	242	177.5	156	246	175	148
DITW097	Kitami 35	Hokkaido	II	*vrn-A1*	*vrn-B1*	*vrn-D1*	*Ppd-A1b*	Hapl-II	Hapl-I	winter	insensitive	235	167.5	146	234	164.5	136
DITW098	Kitakei 1354	Hokkaido	II	*vrn-A1*	*vrn-B1*	*vrn-D1*	*Ppd-A1a*	Hapl-I	Hapl-II	winter	insensitive	244.5	177	166	241.5	175	153.5
DITW099	Kitakei 1660	Hokkaido	II	*vrn-A1*	*vrn-B1*	*vrn-D1*	*Ppd-A1b*	Hapl-I	Hapl-II	winter	sensitive	246.5	184	173	245.5	187	162
DITW100	OW104	Hokkaido	I	*vrn-A1*	[a]NA	*vrn-D1*	*Ppd-A1b*	Hapl-I	Hapl-I	winter	insensitive	236.5	175	154.5	237.5	170.5	145
DITW101	GK Szemes	Hungary	I	*vrn-A1*	*vrn-B1*	*vrn-D1*	*Ppd-A1b*	Hapl-I	Hapl-I	winter	insensitive	244	177.5	164	244	176	153
DITW102	KS831957	USA	I	*vrn-A1*	*vrn-B1*	*vrn-D1*	*Ppd-A1a*	Hapl-I	Hapl-II	winter	insensitive	237.5	173.5	154	237	168.5	142.5
DITW103	Satsukei 159	Hokkaido	I	*vrn-A1*	*vrn-B1*	*vrn-D1*	*Ppd-A1a*	Hapl-I	Hapl-II	winter	insensitive	237.5	169.5	157.5	232	162.5	137.5
DITW104	Kitami 86	Hokkaido	II	*vrn-A1*	*vrn-B1*	*vrn-D1*	*Ppd-A1a*	Hapl-I	Hapl-II	winter	insensitive	239	171	154	236.5	167	146.5
DITW105	Kitami 87	Hokkaido	II	*vrn-A1*	*vrn-B1*	*vrn-D1*	*Ppd-A1a*	Hapl-I	Hapl-II	winter	insensitive	243.5	176.5	159.5	238	175	152
DITW106	Kitami 89	Hokkaido	II	*vrn-A1*	*vrn-B1*	*vrn-D1*	*Ppd-A1a*	Hapl-II	Hapl-II	winter	insensitive	244.5	175	156	240	170	147
DITW107	Kitami 92	Hokkaido	II	*vrn-A1*	*vrn-B1*	*vrn-D1*	*Ppd-A1a*	Hapl-I	Hapl-II	winter	insensitive	240.5	175.5	158.5	238.5	170	149.5
DITW108	Kitami 93	Hokkaido	II	*vrn-A1*	*vrn-B1*	*vrn-D1*	*Ppd-A1a*	Hapl-I	Hapl-II	winter	insensitive	242	175	157	238	170	149
DITW109	Kitami 95	Hokkaido	II	*vrn-A1*	*vrn-B1*	*vrn-D1*	*Ppd-A1a*	Hapl-I	Hapl-II	winter	insensitive	242	175	156.5	239	172.5	147.5
DITW110	Kitami 96	Hokkaido	I	*vrn-A1*	*vrn-B1*	*vrn-D1*	*Ppd-A1a*	Hapl-I	Hapl-I	winter	insensitive	239.5	170	152	235.5	166.5	138
DITW111	Kitamiharu 78	[b]Hokkaido (S)	I	*Vrn-A1a*	*Vrn-B1a*	*Vrn-D1a*	*Ppd-A1b*	Hapl-I	Hapl-II	spring	sensitive	[a]NA	174.5	152	[a]NA	168.5	141.5
DITW112	Kitamiharu 79	[b]Hokkaido (S)	I	*Vrn-A1a*	*Vrn-B1a*	*Vrn-D1a*	*Ppd-A1b*	Hapl-I	Hapl-I	spring	insensitive	238.5	166.5	138.5	[a]NA	161.5	132.5
DITW113	Kitakei 1917	Hokkaido	II	*vrn-A1*	*vrn-B1*	*vrn-D1*	*Ppd-A1a*	Hapl-I	Hapl-I	winter	insensitive	235	167.5	145.5	234	161	136.5
DITW114	Kitakei 1931	Hokkaido	II	*vrn-A1*	*vrn-B1*	*vrn-D1*	*Ppd-A1a*	Hapl-I	Hapl-II	winter	insensitive	234	168	153.5	234	165.5	143
DITW115	Hokkai 265	Hokkaido	I	*vrn-A1*	*vrn-B1*	*vrn-D1*	*Ppd-A1a*	Hapl-II	Hapl-I	winter	insensitive	239	171.5	152	237.5	167.5	140.5
DITW116	Noken komugi 2	Kanto/Tosan/Tokai	IV	*vrn-A1*	*vrn-B1*	*Vrn-D1b*	*Ppd-A1b*	Hapl-V	Hapl-I	spring	insensitive	226.5	154	125	226	150.5	124
DITW117	Noken komugi 3	Kanto/Tosan/Tokai	IV	*vrn-A1*	*vrn-B1*	*Vrn-D1a*	*Ppd-A1b*	Hapl-I	Hapl-I	spring	insensitive	222.5	154	127.5	228	151	126.5

DITW118	Kanto 143	Kanto/Tosan/Tokai	II	*vrn-A1*	*vrn-B1*	*Vrn-D1a*	*Ppd-A1a*	Hapl-I	Hapl-I	spring	insensitive	225	155	129	231.5	154	128.5
DITW119	Kanto 142	Kanto/Tosan/Tokai	IV	*vrn-A1*	*vrn-B1*	*Vrn-D1a*	*Ppd-A1b*	Hapl-I	Hapl-I	spring	insensitive	225	154	130.5	[a]NA	153	127.5
DITW120	TY714-10	Kanto/Tosan/Tokai	III	*vrn-A1*	*vrn-B1*	*Vrn-D1a*	*Ppd-A1b*	Hapl-I	Hapl-I	spring	insensitive	230	159.5	133.5	231.5	156.5	131
DITW121	Kanto 137	Kanto/Tosan/Tokai	IV	*vrn-A1*	*vrn-B1*	*Vrn-D1b*	*Ppd-A1b*	Hapl-I	Hapl-I	spring	insensitive	227.5	155	130.5	230.5	154.5	128.5
DITW122	Tanikei sho RB5008-3-3	Kanto/Tosan/Tokai	IV	*Vrn-A1a*	*Vrn-B1a*	*Vrn-D1a*	*Ppd-A1b*	Hapl-V	Hapl-I	spring	insensitive	226	155.5	120.5	[a]NA	150.5	121
DITW123	Chogokuwase	Kanto/Tosan/Tokai	IV	*vrn-A1*	[a]NA	*Vrn-D1a*	*Ppd-A1b*	Hapl-V	Hapl-I	spring	insensitive	222	138	116	[a]NA	138	108.5
DITW125	Chugoku 168	Kinki/Chugoku/Shikoku	IV	*vrn-A1*	*vrn-B1*	*Vrn-D1a*	*Ppd-A1b*	Hapl-I	Hapl-I	spring	insensitive	[a]NA	152	121	[a]NA	147.5	126
DITW126	Setokirara	Kinki/Chugoku/Shikoku	IV	*vrn-A1*	*vrn-B1*	*Vrn-D1a*	*Ppd-A1b*	Hapl-I	Hapl-I	spring	insensitive	223.5	156	125	[a]NA	149.5	127
DITW127	Saikai 190	Kyushu	IV	*vrn-A1*	*vrn-B1*	*Vrn-D1a*	*Ppd-A1b*	Hapl-V	Hapl-I	spring	insensitive	223.5	152.5	121	228	148	123
DITW128	Saikai 199	Kyushu	IV	*vrn-A1*	*vrn-B1*	*Vrn-D1a*	*Ppd-A1b*	Hapl-I	Hapl-I	spring	insensitive	227	156	129	229	151.5	130
DITW129	Saikai 202	Kyushu	IV	*vrn-A1*	*vrn-B1*	*Vrn-D1a*	*Ppd-A1b*	Hapl-I	Hapl-I	spring	insensitive	234	158	130	[a]NA	153.5	129.5
DITW130	Saikai 203	Kyushu	IV	*vrn-A1*	*vrn-B1*	*Vrn-D1a*	*Ppd-A1b*	Hapl-I	Hapl-I	spring	insensitive	224.5	156	130.5	[a]NA	151	129
DITW131	Saikai 204	Kyushu	IV	*Vrn-A1a*	*vrn-B1*	*Vrn-D1a*	*Ppd-A1b*	Hapl-I	Hapl-I	spring	insensitive	227.5	159.5	125.5	[a]NA	150	125.5
DITW132	Saikai 205	Kyushu	IV	*vrn-A1*	*vrn-B1*	*Vrn-D1a*	*Ppd-A1b*	Hapl-I	Hapl-I	spring	insensitive	225	154.5	126	[a]NA	151.5	127.5
DITW133	Hakei W 1380	Kyushu	IV	*vrn-A1*	*vrn-B1*	*Vrn-D1a*	*Ppd-A1b*	Hapl-I	Hapl-I	spring	insensitive	228.5	155.5	134.5	[a]NA	152.5	135
DITW134	Hakei W 1320	Kyushu	II	*vrn-A1*	*vrn-B1*	*Vrn-D1a*	*Ppd-A1a*	Hapl-V	Hapl-II	spring	insensitive	227	155.5	129	229	150.5	130.5
DITW135	Hakei W 1318	Kyushu	IV	*vrn-A1*	*vrn-B1*	*Vrn-D1a*	*Ppd-A1a*	Hapl-V	Hapl-II	spring	insensitive	226.5	155	129.5	[a]NA	151.5	129.5
DITW136	Norin 61	Kyushu	III	*vrn-A1*	*vrn-B1*	*Vrn-D1a*	*Ppd-A1b*	Hapl-V	Hapl-I	spring	insensitive	229	164.5	130.5	[a]NA	157.5	132

[a]NA: Not available

[b](S): Spring-sowing varieties

Supplemental Table 2. Summary of locations and sowing date

locations	Institution	longitude (N)	latitude (E)	Sowing date (2019-2020)	Sowing date (2020-2021)
Morioka	Tohoku Agricultural Research Center	39.74	141.13	September 25	September 24
Tsukuba	Institution of Crop Science	36.03	140.01	Nobember 7	Nobember 5
Chikugo	Kyushu-Okinawa Agricultural Research Center	33.21	130.49	Nobember 13	November 17

Supplemental Table 3. List of primer sets used for genotyping the *Vrn-1* and *Ppd-1* genes

Gene	Allele or Haplotype	Primer name	primer (5'-3')	Product size (bp)	Annealing temperature (˚C)	Reference
Vrn-A1	vrn-A1/Vrn-A1a/Vrn-A1b/Vrn-A1c	VRN1AF	GAAAGGAAAAATTCTGCTCG	Vrn-A1a: 950 and 876<br>vrn-A1/Vrn-A1b/Vrn-A1c: 714 or 734	55	Yan *et al.* 2004b
		VRN1R	GCAGGAAATCGAAATCGAAG			
	Vrn-A1b	Vrn-P2	CCTGCCGGAATCCTCGTTTT	Vrn-A1b (deletion in promoter): 147<br>no deletion: 167	63	Chen *et al.* 2013
		Vrn-P2	CTACGCCCCTACCCTCCAACA			
	Vrn-A1c	Intrl/A/F2	AGCCTCCACGGTTTGAAAGTAA	1170	65	Fu *et al.* 2005
		Intrl/A/R3	AAGTAAGACAACACGAATGTGAGA			
	vrn-A1	Intrl/C/F	GCACTCCTAACCCACTAACC	no deletion in 1st intron: 1068	59	Fu *et al.* 2005
		Intrl/AB/R	TCATCCATCATCAAGGCAAA			
Vrn-B1	Vrn-B1a	Intrl/B/F	CAAGTGGAACGGTTAGGACA	709	58	Fu *et al.* 2005
		Intrl/B/R3	CTCATGCCAAAAATTGAAGATGA			
	vrn-B1	Intrl/B/F	CAAGTGGAACGGTTAGGACA	1150	59	Fu *et al.* 2005
		Intrl/B/R4	CAAATGAAAAGGAATGAGAGCA			
	Vrn-B1b	Vrn-P7	CCAATCTCACATGCCTCCAA	Vrn-B1b (deletion in 1st intron): 215<br>no deletion: 252	59	Chen *et al.* 2013
		Vrn-P7	ATGCGCCATGAACAACAAAG			
Vrn-D1	Vrn-D1	Intrl/D/F	GTTGTCTGCCTCATCAAATCC	deletion in 1st intron: 1671	65	Fu *et al.* 2005
		Intrl/D/R3	GGTCACTGGTGGTCTGTGC			
	Vrn-D1a	VRN1DF	CGACCCGGGCGGCACGAGTG	612	65	Zhang *et al.* 2012
		VRN1-SNP161CR	AGGATGGCCAGGCCAAAACG			
	Vrn-D1b	VRN1DF	CGACCCGGGCGGCACGAGTG	612	65	Zhang *et al.* 2012
		VRN1-SNP161AR	AGGATGGCCAGGCCAAAACT			
Ppd-A1	Ppd-A1a/Ppd-A1b	Ppd-A1F	CGTACTCCCTCCGTTTCTTT	Ppd-A1a: 338<br>Ppd-A1b: 299	57	Nishida *et al.* 2013
		Ppd-A1-R2	AATTTACGGGGACCAAATACC			
		Ppd-A1-R3	GTTGGGGTCGTTTGGTGGTG			
Ppd-B1 *	Ppd-B1a	Ppd-B1-F	CAGCTCCTCCGTTTGCTTCC	Ppd-B1a: 620<br>Ppd-B1b: 312	60	Nishida *et al.* 2013
		Ppd-B1-R	CAGAGGAGTAGTCCGCGTGT			
	Truncated Ppd-B1 gene in the ‘Chinese Spring’ allele	Ppd-B1c-F-T	TAACTGCTCCTCACAAGTGC	425	65	Beales *et al.* 2007<br>Chen *et al.* 2013
		Ppd-B1c-R-T	CCGGAACCTGAGGATCATC			
	Intact Ppd-B1 copies in the ‘Chinese Spring’ allele	Ppd-B1c-F-I	AAAACATTATGCATATAGCTTGTGTC	994	61	Beales *et al.* 2007<br>Chen *et al* . 2013
		Ppd-B1c-R-I	CAGACATGGACTCGGAACAC			
	Intact Ppd-B1 copies in the ‘Sonora64’/ ‘Timstein’ allele	Ppd-B1a-F	CCAGGCGAGTGATTTACACA	223	60	Diaz *et al.* 2012<br>Chen *et al.* 2013
		Ppd-B1a-R	GGGCACGTTAACACACCTTT			
Ppd-D1 **	2089 bp deletion in promoter (Ppd-D1a)	Ppd-D1a-F	ACGCCTCCCACTACACTG	288	54	Beales *et al.* 2007<br>Chen *et al.* 2013
		Ppd-D1a-R	CACTGGTGGTAGCTGAGATT			
	5 bp deletion in Exon 7 (Ppd-D1d)	Ppd-D1d-F	GTGTCCTTTGCGAATCCTT	deletion: 179<br>no deletion: 184	55	Guo *et al.* 2010<br>Chen *et al.* 2013
		Ppd-D1d-R	TTGGAGCCTTGCTTCATCT			
	TE insertion in Intron 1 (Ppd-D1c)	Ppd-D1c-F	AGGTCCTTACTCATACTCAATCTCA	no TE insertion: 2612	61	Guo *et al.* 2010<br>Chen *et al* . 2013
		Ppd-D1c-R	CTCCCATTGTTGGTGTTGTTA			
	16 bp insertion in Exon 8	Ppd-P9-F	GATGAACATGAAACGGG	insertion: 336<br>no insertion: 320	55	Guo *et al.* 2010<br>Chen *et al.* 2013
		Ppd-P9-R	GTCTAAATAGTAGGTACTAGG			

*: Haplotypes of *Ppd-B1* are decided with combination of genotypes by three primer sets for CNV according to Zhang *et al.* (2015).

**: Haplotypes of *Ppd-D1* are decided with combination of genotypes by four primer sets according to Zhang *et al.* (2015).

Díaz, A., M. Zikhali, A.S. Turner, P. Isaac and D.A. Laurie (2012) Copy number variation affecting the *Photoperiod-B1* and *Vernalization-A1* genes is associated with altered flowering time in wheat (*Triticum aestivum*). PLoS ONE 7: e33234.

Guo, Z., Y. Song, R. Zhou, Z. Ren and J. Jia (2010) Discovery, evaluation and distribution of haplotypes of the wheat *Ppd-D1* gene. New Phytol. 185: 841–851.

Supplemental Table 4. Summary of allele frequency, population, vernalization requirement and photoperiod sensitivity in six breeding areas

		Hokkaido	[a]Hokkaido (S)	Tohoku/Hokuriku	Kanto/Tosan/Tokai	Kinki/Chugoku/Shikoku	Kyushu
population							
	I	11	10	12	5	0	0
	II	19	0	0	1	1	1
	III	0	0	0	11	3	2
	IV	0	0	3	17	8	24
allele							
Vrn-A1	***Vrn-A1a***	0	10	0	3	0	2
	Vrn-A1b	0	0	0	0	0	0
	vrn-A1	30	0	15	31	12	25
Vrn-B1	***Vrn-B1a***	0	8	0	2	0	0
	vrn-B1	29	0	11	31	12	27
Vrn-D1	***Vrn-D1a***	0	3	0	23	12	23
	Vrn-D1b	0	0	0	6	0	3
	vrn-D1	30	7	15	5	0	1
Ppd-A1	***Ppd-A1a***	19	0	0	1	1	2
	Ppd-A1b	11	10	15	33	11	25
Ppd-B1	***Ppd-B1a***	0	0	0	0	1	1
	Hapl-I	23	6	5	21	7	15
	Hapl-II	7	4	6	1	1	0
	**Hapl-V**	0	0	4	12	3	11
Ppd-D1	**Hapl-I**	10	1	15	34	12	25
	Hapl-II	18	7	0	0	0	2
	Hapl-III	2	2	0	0	0	0
venalization requirement							
	winter	30	0	15	4	0	0
	spring	0	10	0	30	12	27
photoperiod sensitivity							
	sensitive	6	9	0	0	0	0
	insensitive	24	1	15	34	12	27

The spring alleles of *Vrn-1* and insensitive allele of *Ppd-1* are shown in bold

[a](S): Spring-sowing varieties

Supplemental Table 5. Distribution of haplotype combinations of *Vrn-1* and *Ppd-1* homoeologues in the 134 varieties

Haplotype							Number of varieties					Days to heading					
Vrn-A1	*Vrn-B1*	*Vrn-D1*	*Ppd-A1*	*Ppd-B1*	*Ppd-D1*	[b]V/P	Population I	Population II	Population III	Population IV	Total	Morioka (19-20)	Tsukuba (19-20)	Chikugo (19-20)	Morioka (20-21)	Tsukuba (20-21)	Chikugo (20-21)
vrn-A1	*vrn-B1*	***Vrn-D1a***	*Ppd-A1b*	Hapl-I	**Hapl-I**	S/I	0	0	6	25	31	225.5	155.8	128.7	230.4	152.5	128.9
vrn-A1	*vrn-B1*	***Vrn-D1a***	*Ppd-A1b*	**Hapl-V**	**Hapl-I**	S/I	2	0	6	10	18	226.7	159.6	131.0	231.0	154.4	129.9
vrn-A1	*vrn-B1*	*vrn-D1*	***Ppd-A1a***	Hapl-I	Hapl-II	W/I	2	11	0	0	13	240.8	174.4	158.7	238.5	170.8	148.1
vrn-A1	*vrn-B1*	*vrn-D1*	*Ppd-A1b*	Hapl-I	**Hapl-I**	W/I	7	0	1	0	9	238.7	172.5	155.5	237.9	169.7	144.6
vrn-A1	*vrn-B1*	*vrn-D1*	*Ppd-A1b*	Hapl-II	**Hapl-I**	W/I	5	1	0	0	6	233.8	167.3	147.3	233.4	164.3	138.3
vrn-A1	*vrn-B1*	*vrn-D1*	*Ppd-A1b*	**Hapl-V**	**Hapl-I**	W/I	1	0	1	3	5	233.2	167.1	146.0	232.5	163.3	136.8
vrn-A1	*vrn-B1*	***Vrn-D1b***	*Ppd-A1b*	Hapl-I	**Hapl-I**	S/I	1	0	1	3	5	228.1	156.7	133.8	229.1	155.4	130.2
vrn-A1	*vrn-B1*	*vrn-D1*	*Ppd-A1b*	Hapl-I	Hapl-II	W/S	1	2	1	0	4	244.5	180.3	168.1	242.8	180.8	158.6
vrn-A1	*vrn-B1*	***Vrn-D1b***	*Ppd-A1b*	**Hapl-V**	**Hapl-I**	S/I	0	0	1	3	4	225.8	155.3	127.6	227.0	151.8	126.6
vrn-A1	[a]NA	*vrn-D1*	*Ppd-A1b*	Hapl-I	**Hapl-I**	S/I	3	0	0	0	3	235.3	170.7	150.2	234.7	165.8	140.5
vrn-A1	*vrn-B1*	*vrn-D1*	***Ppd-A1a***	Hapl-II	**Hapl-I**	S/I	2	1	0	0	3	240.5	172.2	152.3	236.7	167.0	139.8
Vrn-A1a	***Vrn-B1a***	*vrn-D1*	*Ppd-A1b*	Hapl-II	Hapl-II	S/I	3	0	0	0	3	237.0	176.5	158.0	241.0	169.5	149.0
Vrn-A1a	*vrn-B1*	*vrn-D1*	*Ppd-A1b*	Hapl-I	**Hapl-I**	S/I	1	0	0	1	2	225.0	158.3	132.3	228.5	155.0	132.3
vrn-A1	*vrn-B1*	***Vrn-D1a***	***Ppd-A1a***	Hapl-I	**Hapl-I**	S/I	0	2	0	0	2	224.3	153.8	127.3	231.5	151.0	127.0
vrn-A1	*vrn-B1*	*vrn-D1*	***Ppd-A1a***	Hapl-I	**Hapl-I**	S/I	1	1	0	0	2	237.3	168.8	148.8	234.8	163.8	137.3
vrn-A1	*vrn-B1*	*vrn-D1*	*Ppd-A1b*	Hapl-I	Hapl-III	S/S	2	0	0	0	2	243.3	184.5	173.8	243.5	183.0	164.0
Vrn-A1a	***Vrn-B1a***	***Vrn-D1a***	*Ppd-A1b*	Hapl-I	Hapl-II	S/I	2	0	0	0	2	242.5	176.3	156.8	NA	172.0	146.8
vrn-A1	*vrn-B1*	*vrn-D1*	***Ppd-A1a***	Hapl-II	Hapl-II	S/I	0	2	0	0	2	243.3	176.0	158.0	240.3	171.8	148.0
vrn-A1	[a]NA	*vrn-D1*	*Ppd-A1b*	Hapl-II	**Hapl-I**	S/I	2	0	0	0	2	233.5	165.3	144.0	232.5	160.8	134.8
vrn-A1	*vrn-B1*	***Vrn-D1a***	***Ppd-A1a***	**Hapl-V**	Hapl-II	S/I	0	0	0	2	2	226.8	155.3	129.3	229.0	151.0	130.0
vrn-A1	*vrn-B1*	***Vrn-D1a***	*Ppd-A1b*	***Ppd-B1a***	**Hapl-I**	S/I	0	0	1	1	2	221.5	149.8	120.8	226.0	146.8	121.8
Vrn-A1a	***Vrn-B1a***	*vrn-D1*	*Ppd-A1b*	Hapl-I	**Hapl-I**	S/I	0	0	0	1	1	227.0	155.5	128.5	NA	152.5	128.5
Vrn-A1a	*vrn-B1*	***Vrn-D1a***	*Ppd-A1b*	Hapl-I	**Hapl-I**	S/I	0	0	0	1	1	227.5	159.5	125.5	NA	150.0	125.5
Vrn-A1a	[a]NA	*vrn-D1*	*Ppd-A1b*	Hapl-I	Hapl-II	S/S	1	0	0	0	1	246.5	185.0	170.5	245.5	182.0	164.0
Vrn-A1a	[a]NA	*vrn-D1*	*Ppd-A1b*	Hapl-I	Hapl-III	S/I	1	0	0	0	1	236.0	175.5	155.5	244.0	169.5	150.0
Vrn-A1a	***Vrn-B1a***	*vrn-D1*	*Ppd-A1b*	Hapl-I	Hapl-II	S/I	1	0	0	0	1	242.0	179.5	165.0	242.5	178.0	157.0
Vrn-A1a	***Vrn-B1a***	*vrn-D1*	*Ppd-A1b*	Hapl-I	Hapl-III	S/I	1	0	0	0	1	246.5	184.0	171.5	NA	185.0	163.0
Vrn-A1a	***Vrn-B1a***	***Vrn-D1a***	*Ppd-A1b*	Hapl-I	**Hapl-I**	S/I	1	0	0	0	1	238.5	166.5	138.5	NA	161.5	132.5
Vrn-A1b	***Vrn-B1a***	*vrn-D1*	*Ppd-A1b*	Hapl-I	**Hapl-I**	S/I	1	0	0	0	1	242.5	174.5	144.0	241.5	164.0	138.0
vrn-A1	*vrn-B1*	***Vrn-D1a***	*Ppd-A1b*	Hapl-II	**Hapl-I**	S/I	0	0	0	1	1	224.0	154.0	122.5	229.0	152.5	128.0
vrn-A1	*vrn-B1*	*vrn-D1*	*Ppd-A1b*	Hapl-II	Hapl-II	S/S	0	1	0	0	1	238.5	175.5	164.0	239.5	171.5	149.0
Vrn-A1a	***Vrn-B1a***	***Vrn-D1a***	*Ppd-A1b*	**Hapl-V**	**Hapl-I**	S/I	0	0	0	1	1	226.0	155.5	120.5	NA	150.5	121.0
vrn-A1	[a]NA	***Vrn-D1a***	*Ppd-A1b*	**Hapl-V**	**Hapl-I**	S/I	0	0	0	1	1	222.0	138.0	116.0	NA	138.0	108.5
vrn-A1	*vrn-B1*	***Vrn-D1a***	*Ppd-A1b*	**Hapl-V**	Hapl-II	S/I	0	0	1	0	1	242.0	177.5	156.0	246.0	175.0	148.0

[a]NA: Not available

[b]V/P: vernalization requirement/photoperiod sensitibity (W:winter, S:spring/I:insensitive, S:sensitive)

The spring alleles of *Vrn-1* and insensitive allele of *Ppd-1* are shown in bold

Supplemental Table 6. Two-way ANOVA results on the influence of vernalization requirement, photoperiod sensitivity and their interaction on thedays to heading in six environments

		dgree of freedom	sum of square	mean sum of square	F value
Moroika (19-20)					
	vernalization requirement (V)	1	2464.4	2464.4	92.4***
	photoperiod sensitivity (P)	1	1460.9	1460.9	54.7***
	V X P	1	186.5	186.5	7.0**
	Residuals	123	3282.0	26.7	
Tsukuba (19-20)					
	vernalization requirement (V)	1	3599.6	3599.6	87.9***
	photoperiod sensitivity (P)	1	4059.2	4059.2	99.1***
	V X P	1	272.2	272.2	6.6*
	Residuals	130	5324.1	41.0	
Chikugo (19-20)					
	vernalization requirement (V)	1	11591.8	11591.8	141.6***
	photoperiod sensitivity (P)	1	8448.8	8448.8	103.2***
	V X P	1	251.8	251.8	3.1
	Residuals	130	10643.8	81.9	
Moroika (20-21)					
	vernalization requirement (V)	1	242.9	242.9	11.5**
	photoperiod sensitivity (P)	1	847.0	847.0	40.2***
	V X P	1	46.0	46.0	2.2
	Residuals	93	1961.1	21.1	
Tsukuba (20-21)					
	vernalization requirement (V)	1	4281.2	4281.2	112.5***
	photoperiod sensitivity (P)	1	4351.0	4351.0	114.3***
	V X P	1	89.0	89.0	2.3
	Residuals	130	4948.0	38.1	
Chikugo (20-21)					
	vernalization requirement (V)	1	3957.5	3957.5	81.9***
	photoperiod sensitivity (P)	1	5520.6	5520.6	114.3***
	V X P	1	21.6	21.6	0.45
	Residuals	130	6281.6	48.3	
